# DNA methylome-wide alterations associated with estrogen receptor-dependent effects of bisphenols in breast cancer

**DOI:** 10.1186/s13148-019-0725-y

**Published:** 2019-10-10

**Authors:** Z. Awada, R. Nasr, R. Akika, V. Cahais, C. Cuenin, M. Zhivagui, Z. Herceg, A. Ghantous, N. K. Zgheib

**Affiliations:** 1 Pharmacology and Toxicology, Faculty of Medicine, American University of Beirut, Riad El-Solh, Beirut, 1107-2020 Lebanon; 2 Anatomy, Cell Biology and Physiology, Faculty of Medicine, American University of Beirut, Beirut, Lebanon; 3 Epigenetics group, International Agency for Research on Cancer, Cours Albert Thomas, 69372 Lyon, France

**Keywords:** Endocrine disruptor, BPA, BPF, BPS, Breast cancer, DNA methylation

## Abstract

**Background:**

Bisphenol A (BPA), an estrogen-like endocrine disruptor used in plastics, has been associated with development and promotion of breast cancer, so plastic manufacturers shifted towards less-studied analogs, BPF and BPS. Studying the associated DNA methylome-wide mechanisms of these derivatives is timely, particularly in comparison with BPA.

**Methods:**

We assessed proliferation, cell cycle, and migration of breast cancer cells (estrogen receptor (ER)-positive: MCF-7 and ER-negative: MDA-MB-231) treated with BPF and BPS ± estrogen receptor inhibitor (ERI) in comparison to BPA ± ERI. RNA expression and activity of DNA (de)methylation enzymes and *LINE-1* methylation were quantified. DNA methylome-wide analysis was evaluated in bisphenol-exposed cells and compared to clinical breast cancer data.

**Results:**

The three bisphenols caused ER-dependent increased proliferation and migration of MCF-7 but not MDA-MB-231 cells, with BPS being 10 times less potent than BPA and BPF. Although they have similar chemical structures, the three bisphenols induced differential DNA methylation alterations at several genomic clusters of or single CpG sites, with the majority of these being ER-dependent. At equipotent doses, BPA had the strongest effect on the methylome, followed by BPS then BPF. No pathways were enriched for BPF while BPA- and BPS-induced methylome alterations were enriched in focal adhesion, cGMP-PKG, and cancer pathways, which were also dysregulated in methylome-wide alterations comparing ER-positive breast cancer samples to adjacent normal tissues.

**Conclusions:**

The three bisphenols have important epigenetic effects in breast cell lines, with those of BPA and BPS overlapping with cancer-related pathways in clinical breast cancer models. Hence, further investigation of their safety is warranted.

**Electronic supplementary material:**

The online version of this article (10.1186/s13148-019-0725-y) contains supplementary material, which is available to authorized users.

## Introduction

Currently, around two million women suffer from breast cancer worldwide, and this number is expected to increase by 970,000 in 2040 [1]. This phenomenon parallels the increased exposure to environmental toxicants such as endocrine disrupting compounds (EDCs), though a multitude of other factors may play a role [2, 3]. Bisphenol A (BPA), a monomer of polycarbonate plastics and epoxy resins and an EDC with estrogenic activity, has been associated with development and promotion of breast cancer in multiple animal and in vitro models [4–9] and has been recommended with “high priority” for the evaluation of its carcinogenic effects by 2019 by the International Agency for Research on Cancer (IARC) monographs [10].

Given the importance of epigenetics in acting as a molecular sensor to environmental exposures [11], the epigenetic mechanisms of BPA and particularly BPA-induced DNA methylated aberrations have been demonstrated in a number of cell lines [12, 13]. However, only one recent methylome-wide analysis studied the DNA methylation aberrations induced by BPA on MCF-7 breast cells and reported that BPA results in hypermethylation of tumor suppressor genes and hypomethylation of oncogenes [14]. Of interest, DNA methylation aberrations are considered the hallmark of cancer development, and similarly to other cancers, hypermethylation of tumor suppressor genes and global DNA hypomethylation were reported in breast cancer [15–18].

Because of the health concerns of BPA use, manufacturers replaced it with structural analogs, BPF, and BPS (Additional file 1: Figure S1); however, compared to BPA, there is limited knowledge of their cancer-related potential and underlying mechanisms in the breast. A recent in vitro study showed that, similarly to BPA, BPF and BPS increased the proliferation and migration of breast carcinoma cells (MCF-7 CV) and increased the protein expression of cell cycle progression genes and markers of epithelial to mesenchymal transition [19]. As for their mechanism of action, most of the studies focused on the estrogenic activity of these analogs [20, 21], and a systematic review showed that BPF has a comparable estrogenic activity to BPA, while BPS is about 10 times less potent than BPA and BPF [21]. The DNA methylome-wide effects of these analogs remain to be addressed.

In this study, we aimed to evaluate the DNA methylome-wide alterations, including estrogen receptor (ER) dependence and associated mechanisms, of BPF and BPS in comparison with BPA in breast cancer cells. We also aimed to compare the genome-wide methylation results with those dysregulated in ER-positive breast cancer tissues.

## Methods

MCF-7 (ER-positive) and MDA-MB-231 (ER-negative) breast cancer cell lines were treated with BPA, BPF, and BPS ranging from non-occupational environmental exposure doses (10^−8^ M for BPA and BPF; 10^−9^ M for BPS) to high dose (10^−4^ M) in the presence and absence of ERI for 24, 48, and 72 h, and assessed for cell metabolic activity and viability using MTT and trypan blue assay, respectively. Non-occupational human exposure doses (10^−8^ M for BPA and BPF; 10^−9^ M for BPS) and minimum functional doses (10^−6^ M for BPA and BPF; 10^−5^ M for BPS), which noteworthy for BPA approached its occupational doses [22], were used in cell cycle, cell scratch, cell morphology, and molecular assays. These included reverse transcription-polymerase chain reaction (RT-PCR) measurement of the gene expression of DNA (de)methylation enzymes, measurement of the activity of DNA methylation and demethylation enzymes, *LINE-1* pyrosequencing, and methylome-wide profiling using Infinium MethylationEPIC microarrays. Bisphenol-induced differentially methylated genes were compared with those differentially methylated in ER-positive breast cancer patients relative to adjacent normal tissue from The Cancer Genome Atlas (TCGA) database.

### Bisphenol reagents and related chemicals

BPA (cat#239658), BPF (cat# 51453), and BPS (cat# 43034) were purchased from Sigma-Aldrich (Taufkirchen, Germany), and estrogen receptor inhibitor (ERI) fulvestrant, ICI 182,780 (cat# sc-203435), was purchased from Santa Cruz Biotechnology, Inc. (Dallas, TX, USA). BPA, BPF, and BPS were dissolved in either absolute DMSO (cat# 41640, Sigma-Aldrich, Taufkirchen, Germany) or ethanol (cat# ET0006, Scharlab S.L., Barcelona, Spain) at stock concentrations of 1 M, and ERI was dissolved in absolute DMSO at stock concentration of 100 μM. Stock solutions were stored in aliquots at − 20 °C.

### Choice of doses

Epidemiological studies detected BPA and its analogs BPF and BPS in a large number of plasma and/or urine samples from human individuals [23–28]. Non-occupational plasma and urine levels of BPA ranged roughly from less than the level of detection (LOD) to 9.6 × 10^−8^ M [23–25], but those of BPS were 10 folds lower than BPA [26]. To date, no report is available concerning the plasma level of BPF; however, its urine levels were comparable to those of BPA in epidemiological studies [28, 29]. Hence, we considered plasma and/or urine levels of 10^−8^ M BPA, 10^−8^ M BPF, and 10^−9^ M BPS as human exposure doses and tested them in our study. For selection of the dose that may induce phenotypic and, hence, molecular changes in breast cancer cell lines, doses ranging from 10^−4^ M (very high) to human exposure dose (10^−8^ M for BPA and BPF, 10^−9^ M for BPS) were tested in MTT (3-(4,5-dimethylthiazol-2-yl)-2,5-diphenyltetrazolium bromide) and trypan blue assays. The human exposure dose, together with the minimum functional dose that was associated with marked increase in cell metabolic activity and viability were then tested for cell cycle distribution, cell migration, and cell morphology.

### Cell culture and media

MCF-7 (ER-positive) and MDA-MB-231 (ER-negative) cell lines originating from human breast epithelial adenocarcinomas were obtained from the American Type Culture Collection (ATCC, Manassas, VA, USA). They were cultured in Dulbecco’s modified Eagle’s medium (DMEM) (cat# BE-12-741F, Lonza, Basel, Switzerland) supplemented with 10% fetal bovine serum (FBS) (cat# F2442, Sigma-Aldrich, Taufkirchen, Germany), 1% penicillin/streptomycin (cat# 17-602E, Lonza, Basel, Switzerland), and 1% sodium pyruvate (cat# S8636, Sigma-Aldrich, Taufkirchen, Germany) at 37 °C in a humidified atmosphere with 5% CO_2_ and 95% air. Prior to each assay, cells were cultured for 2–3 days in phenol red-free DMEM (cat# BE12-917F, Lonza, Basel, Switzerland) supplemented with 10% charcoal-stripped FBS (cat# F6765, Sigma-Aldrich, Taufkirchen, Germany), 2% L-glutamine (cat# G7513, Sigma-Aldrich, Taufkirchen, Germany), and 1% penicillin/streptomycin, in order to avoid the effects of the estrogenic components of DMEM and FBS. Cells were detached using 0.25% trypsin (cat# BE17-160E, Lonza, Basel, Switzerland) and 0.53 mM ethylenediaminetetraacetic acid (EDTA) (cat# AM9260G, Ambion, Waltham, MA, USA) solution.

### Cell metabolic activity using MTT assay

MTT assay was performed at 24, 48, and 72 h for each treatment concentration with and without ERI. In brief, MCF-7 and MDA-MB-231 cells were seeded in a 96-well plate at a seeding density of 6000 and 4000 cells, respectively. After overnight incubation, cells were treated in triplicates with different doses of BPA (10^−4^, 10^−5^, 10^−6^, 10^−7^, and 10^−8^ M), BPF (10^−4^, 10^−5^, 10^−6^, 10^−7^, and 10^−8^ M), or BPS (10^−4^, 10^−5^, 10^−6^, 10^−7^, 10^−8^, and 10^−9^ M) with or without ERI (100 nM) for 24, 48, and 72 h. Control cells were treated with 0.2% DMSO (BPA control) or both 0.1% ethanol and 0.1% DMSO (BPF and BPS control). After treatment, cells were incubated with 10 μl of MTT reagent for 4 h, which was followed by overnight incubation with 100 μl of solubilizing agent (cat# 11465007001, Sigma-Aldrich, Taufkirchen, Germany). Absorbance was detected using an ELISA plate reader at wavelength of 595 nm. After subtraction of absorbance obtained from wells containing no cells (negative control), results were calculated as percentage of metabolic activity relative to control. Results are presented as mean optical density (OD) or percentage of OD relative to control (% metabolic activity) + standard error of the mean (SEM) of at least three independent trials.

### Cell viability using trypan blue assay

MCF-7 and MDA-MB-231 cells were seeded in a six-well plate at a seeding density of 3 × 10^5^ and 1 × 10^5^ respectively. After overnight incubation, cells were treated with different doses of BPA (10^−4^, 10^−6^, and 10^−8^ M), BPF (10^−4^, 10^−6^, and 10^−8^ M), or BPS (10^−4^, 10^−5^, and 10^−9^ M) with or without ERI for 24, 48, and 72 h. Control cells were treated with 0.1% DMSO (BPA control) or both 0.1% DMSO and 0.001% ethanol (BPF and BPS control). After treatment, cells were counted using a hematocytometer using trypan blue dye (cat# T8154, Sigma-Aldrich, Taufkirchen, Germany). Results are presented as mean viable cell count or percentage of viable cell count relative to control + SEM of at least three independent trials.

### Cell migration using cell scratch assay

MCF-7 and MDA-MB-231 cells were seeded into six-well plates at a seeding density of 1.5 × 10^−6^ and 1 × 10^−6^ cells, respectively, so that a confluent monolayer is obtained after overnight incubation. Cells were treated with ERI (100 nM) added at least half an hour before treatment. Cell layer was scratched with a 10-μl filter tip, and media were replenished with media containing BPA (10^−6^ and 10^−8^ M), BPF (10^−6^and 10^−8^ M), or BPS (10^−5^ and 10^−9^ M) with or without ERI (100 nM). A particular zone (defined by the intersection between a horizontal line drawn at the other side of the plate and the vertical line of the scratch) was captured by a light microscope at various time points: 0, 2, 4, 6, 8, 10, 12, and 24 h. Cell migration was measured as the distance traveled by the cells, which is the distance between injured cells at time “0” minus the distance between these cells at time “t”. Results are presented as mean distance traveled (arbitrary unit) ± SEM of at least three independent trials.

### Cell cycle analysis using flow cytometry

MCF-7 cells were seeded into six-well plates at a seeding density of 3 × 10^5^ cells. After overnight incubation, cells were treated with BPA (10^−6^ and 10^−8^ M), BPF (10^−6^ and 10^−8^ M), or BPS (10^−5^ and 10^−9^ M) with or without ERI (100 nM) for 24 and 48 h. Control cells were treated with 0.1% DMSO. Cells were washed with ice-cold 1× phosphate-buffered saline (PBS) (cat# BE 17-517Q, Lonza, Basel, Switzerland), fixed with ice-cold absolute ethanol, stained with propidium iodide (cat# P4170, Sigma-Aldrich, Taufkirchen, Germany), and run onto Guava easyCyte Flow Cytometer (Merck KGaA, Darmstadt, Germany). Data of 10,000 cells were collected, and percentage of cells in each cell cycle phase was analyzed using easyCyte software. Results are presented as mean percentage of cells within each cell cycle phase + SEM of at least three independent trials.

### Cell morphology

After 24 and 48 h of treatment with BPA (10^−6^ and 10^−8^ M), BPF (10^−6^ and 10^−8^ M), or BPS (10^−5^ and 10^−9^ M) with or without ERI (100 nM), images of MCF-7 cells were captured using light microscope (Leica DMi1, Leica Microsystems GmbH, Wetzlar, Germany) at × 40 magnification.

### Molecular assays

MCF-7 cells were seeded in six-well plates at a seeding density of 3 × 10^5^ cells. After overnight incubation, cells were treated with BPA (10^−6^ and 10^−8^ M), BPF (10^−6^ and 10^−8^ M), or BPS (10^−5^ and 10^−9^ M) with or without ERI (100 nM) for 24 or 48 h. Control cells were treated with 0.1% DMSO or both 0.1% DMSO and 0.001% ethanol.

### RNA isolation and reverse transcription

RNA was isolated using Trizol-based protocol (Sigma-Aldrich, Taufkirchen, Germany). Isolated RNA samples were treated with DNase using the DNase treatment and removal kit (cat# AM1906, Invitrogen, Waltham, MA, USA) as per manufacturer’s protocol. RNA was then measured using Denovix DS-11 spectrophotometer (Denovix Inc., Wilmington, DE, USA), and both 260/230 and 260/280 ratios were detected for assessment of the purity of samples. RNA samples were then run on an agarose gel to view RNA bands and immediately reverse transcribed to cDNA using the high capacity reverse transcription kit (cat# 4368814, Applied Biosystems, Waltham, MA, USA) as per manufacturer’s protocol. cDNA were stored at − 20 °C until further assays.

### RNA expression using reverse transcription-polymerase chain reaction

RNA expression of DNA methyltransferase 1 (DNMT1), DNA methyltransferase 3a (DNMT3a), DNA methyltransferase 3b (DNMT3b), ten-eleven translocation 1 (TET1), ten-eleven translocation 2 (TET2), and ten-eleven translocation 3 (TET3) was measured using reverse transcription-polymerase chain reaction (RT-PCR). In brief, PCR was performed in a 384-well plate using cDNA template equivalent to 50 ng RNA, 1× SYBR Green master mix (cat# 1708882, Bio-Rad, Hercules, CA, USA) and primers are shown in Additional file 2: Table S1 (each at a final concentration of 100 nM). No template control (NTC) and no amplification control (NAC) were run with the samples. PCR thermal cycling conditions were 50 °C for 2 min, 95 °C for 10 min, and 40 cycles of 95 °C for 15 s and 60 °C for 1 min. Each sample was measured in triplicate, and RNA expression of target genes was calculated relative to β2-microglobulin using the 2^−ΔΔct^ method.

A standard curve was generated using serial dilutions (0.05, 0.5, 5, 50 ng) of an RNA sample, and delta threshold cycle (ct) (ct target gene – ct endogenous control) was calculated and plotted versus log (RNA input amount). The standard curve was ideal with an *r*^2^ approaching 1 for all tested genes.

### Protein isolation and quantification

Proteins were isolated using 3[(cholamidopropyl)-dimethyl-ammonium]-1-propanesulfonate (CHAPS) lysis buffer (0.5% CHAPS (cat# ab141396, Abcam, Cambridge, MA, USA), 10 mM Tris-HCl (pH 7.5) (cat# 161-0719, Bio-Rad, Hercules, CA, USA), 1 mM MgCl2 (cat# M-2670, Sigma-Aldrich, Taufkirchen, Germany), 1 mM EGTA (cat# E-4378, Sigma-Aldrich, Taufkirchen, Germany), 5 mM β-mercaptoethanol (cat# 161-0710, Bio-Rad, Hercules, CA, USA), 0.1 mM [4(2-aminoethyl)-benzenesulfonyl fluoride] hydrochloride (cat# P-7626, Sigma-Aldrich, Taufkirchen, Germany), and 10% glycerol (cat# G5516, Sigma-Aldrich, Taufkirchen, Germany). Proteins were incubated with the lysis buffer for 30 min on ice, and vigorous vortexing was performed every 10-min interval. The lysate was then centrifuged at 14,000 rpm for 30 min at 4 °C, and the supernatant was collected.

Protein quantification was performed using Lowry quantification method. In brief, a serial dilution of bovine serum albumin (BSA) (cat# E588, Amresco, Dublin 15, Ireland) was prepared with concentrations ranging from 0.3 to 1.5 mg/ml. Then, samples and standards were treated with Lowry reagents (cat# 500-015, cat# 500-0113, cat# 500-0114, Bio-Rad, Hercules, CA, USA), shook for 1 min, and incubated for 15 min. The absorbance was read at 750 nm with an ELISA plate reader. A standard curve was drawn using the absorbance and concentrations of BSA, and sample protein concentrations were calculated from the standard curve.

### Measurement of DNMT and TET activity

DNMT and TET activities were measured using EpiQuik DNMT activity/inhibition assay ultra kit (cat# P-3009, Epigentek, Farmingdale, NY, USA) and TET hydroxylase activity quantification kit (cat# ab156912, Abcam, Cambridge, MA, USA), respectively, following manufacturer’s protocol. In brief, fresh protein samples (20–50 μg) were incubated with the substrate and assay buffer for 3 h. Wells were then washed and incubated with the capture antibody for 1 h. After that, wells were washed and incubated with the detection antibody and enhancer solution. Finally, a color developing solution was added. Positive and negative controls were run in each assay. Color absorbance was measured at 450 nm wavelength using ELISA plate reader, and background absorbance was measured at 630 nm wavelength. Enzyme activity was calculated as (OD450 − OD630)/[incubation time (h) × protein amount (mg)].

### DNA isolation, quantification, and bisulfite conversion

DNA was isolated using Flexigene DNA isolation kit from Qiagen (cat # 51206, Hilden, Germany) as per manufacturer’s protocol. DNA was then measured using a Denovix DS-11 spectrophotometer (Denovix Inc., Wilmington, DE, USA), and both 260/230 and 260/280 ratios were detected for assessment of the purity of samples. Bisulfite conversion of portion of the isolated DNA was performed using EZ DNA Methylation Kit (Zymo Research, Irvine, CA, USA) as per manufacturer’s protocol. Both bisulfite-converted and non-converted DNA samples were stored at − 20 °C until further assays.

### Bisulfite pyrosequencing of *LINE-1*

*LINE-1* region of interest was amplified on the bisulfite-converted DNA and sequenced as previously described [30, 31] using Hot-Start Taq Master Mix from Qiagen (Hilden, Germany) and the primers listed in Additional file 2: Table S1. Bisulfite pyrosequencing was carried out at IARC on the PyroMark Q96 ID (Qiagen, Hilden, Germany) to measure the methylation percentage of five CpG sites in the *LINE-1* element. Three independent trials were used for bisulfite pyrosequencing of *LINE-1*.

### Whole-genome DNA methylation profiling

Whole-genome DNA methylation analysis was performed at IARC in two independent trials using the Infinium MethylationEPIC microarray that covers over 850,000 CpGs (dinucleotides that are the main target for methylation), following manufacturer’s protocol (Illumina Inc., San Diego, CA, USA). Each chip encompasses eight samples, so we used stratified randomization to mitigate the batch effects, ensuring that matched experimental conditions were present on the same chip and that duplicates were distributed between different chips, when possible. For each sample, 250 ng of bisulfite-converted DNA was used for hybridization on Infinium MethylationEPIC bead arrays, following the manufacturer’s protocol (Illumina Inc., San Diego, CA, USA). Chips were scanned using Illumina iScan to produce two-color raw data files (.idat format).

Raw intensity data files (.idat) were handled in R using the minfi package to calculate the methylation level at each CpG as the beta-value (*β* = intensity of the methylated allele (M)/(intensity of the unmethylated allele (U) + intensity of the methylated allele (M) + 100)), and the data were exported for quality control and processing. Methylation features were filtered from cross-reactive probes and low-quality probes (probes having bead counts < 3 in at least 5% of samples). Data quality was further assessed using box plots for the distribution of methylated and unmethylated signals, and multidimensional scaling plots and unsupervised clustering were used to check for sample outliers, which were removed from the analysis along with samples having > 1% of CpG sites with a detection *p* value > 0.05. The remaining dataset was normalized using the funnorm normalization of the minfi package in order to correct for the technical variability between Probe I and Probe II.

After filtration, density and density bean plots of *β* values showed more homogenous distribution, and density bean plots showed roughly similar median *β* values among the different treatment conditions (Additional file 1: Figure S2). Filtered *β*s were then corrected for different covariates (sample plate, sentrix position, trial number) using surrogate variable analysis (SVA) method and log-transformed to *M* values. Principal component analysis (PCA) for treatment condition and other covariates was performed before and after SVA correction, and no principal component was significantly associated with the outcome indicating that there is no need to adjust for the different components later in the analysis (Additional file 1: Figure S3). Hence, crude robust linear model (RLM) was performed on *M* values to detect statistically significant differentially methylated CpG probes (DMPs) pertaining to different comparisons. Each treatment condition was compared to corresponding control, and adjusted *p* value < 0.05 was considered statistically significant. This model was also performed to detect differentially methylated regions (DMRs) using DMRcate bioinformatics package [32], whereby the genomic DNA is reduced by a dimension reduction technique into clusters of genomically close (within 1 kb distance) and highly correlated CpG sites.

After detecting DMPs and DMRs, these were filtered using the following filtration criteria: for DMPs, mean filtered delta betas (Δβs) ≥ 3%, standard deviations (SDs) of filtered *β*s in treated group < 10%, SDs of *β* values in corresponding control < 10%, and SDs of filtered *β*s in all controls < 10%; while for DMRs: mean Δβfc of significant CpGs within the DMR ≥ 3%, mean filtered Δβ of significant CpGs within the DMR ≥ 3%, mean SD of filtered *β*s in the treated group < 10%, mean SD of filtered *β*s in the corresponding control < 10%, and mean SD of filtered *β*s in all controls < 10%. Venn diagrams showing the number of DMPs and DMRs in different comparisons (BPA/BPF/BPS compared to their control) were drawn using Venny 2.1.0 online tool [33].

We correlated the methylation values of the array versus the pyrosequencing results using three CpG sites each representing one of the three bisphenols BPA, BPF, and BPS. Primers were designed for regions encompassing these CpG sites (Additional file 2: Table S2), and amplicons were analyzed using bisulfite pyrosequencing using the PyroMark Q96 ID (Qiagen, Hilden, Germany). Results of both pyrosequencing and array were correlated using Pearson correlation analysis.

Pathway analysis of the genes with DMPs in the three comparisons (BPA/BPF/BPS compared to their control) was performed using Enrichr (Ma’ayan Laboratory) [34, 35]. Pathways with FDR < 0.05 were considered statistically significant, and a graph was drawn using PRISM software (GraphPad6, La Jolla, CA, USA). Similarly, Venn diagrams showing the number of DMPs in different comparisons (BPA/BPA+ERI/ERI compared to control) were drawn using Venny 2.1.0 online tool [33], and similar figures were done for BPF and BPS. Concerning the DMPs and DMRs selected for the representative figures, these were statistically significant in either BPA or BPF or BPS treatment conditions when compared to control, with high filtered Δβs, and not statistically significant in the ERI group when compared to control. Completely dependent CpGs were statistically significant in neither the BPA + ERI nor BPF + ERI nor BPS + ERI treatment conditions when compared to control; in contrast, completely independent CpGs were statistically significant in these treatment conditions.

### Comparison with TCGA dataset

With the aim to test the relevance of discovered DMPs associated with BPA and its analogs BPF and BPS to breast cancer, we accessed publicly available methylation data of 21,986 CpG sites in 816 breast cancer and 124 adjacent-normal breast tissues of breast cancer patients based on The Cancer Genome Atlas (TCGA) database [17]. Since endocrine disruptors act through ER, analysis was performed on the subset of 595 ER-positive tumor tissues in comparison with the normal tissues using two-tailed *t* test followed by false discovery rate (FDR) correction using Benjamini-Hochberg method (IBM SPSS statistics software, version 24.0, Armonk, NY, USA). An FDR *p* value < 3.15 × 10^−8^ was considered statistically significant, and only CpGs with DNA methylation change (Δβ) ≥ 3% were reported.

### Statistical analysis

Data were analyzed using IBM SPSS software version 24.0 (Armonk, NY, USA) and visualized using GraphPad Prism software version 6 (GraphPad 6, La Jolla, CA, USA). Results of each assay are presented as mean ± SEM of at least three independent trials. For continuous data, comparisons were performed between each treatment condition and control using one-way analysis of variance (ANOVA) followed by Dunnett post hoc test and within the same treatment condition with ERI versus without ERI using ANOVA followed by Tukey’s honestly significant difference (HSD) post hoc test. For cell scratch assay, two-way ANOVA was performed to test for the association of time points and treatment conditions with cell migration. A *p* value of less than 0.05 was considered statistically significant.

## Results

### Bisphenols induced an ER-dependent increase in cell proliferation, migration, and S-G2/M cell cycle proportions

**Fig. 1:**
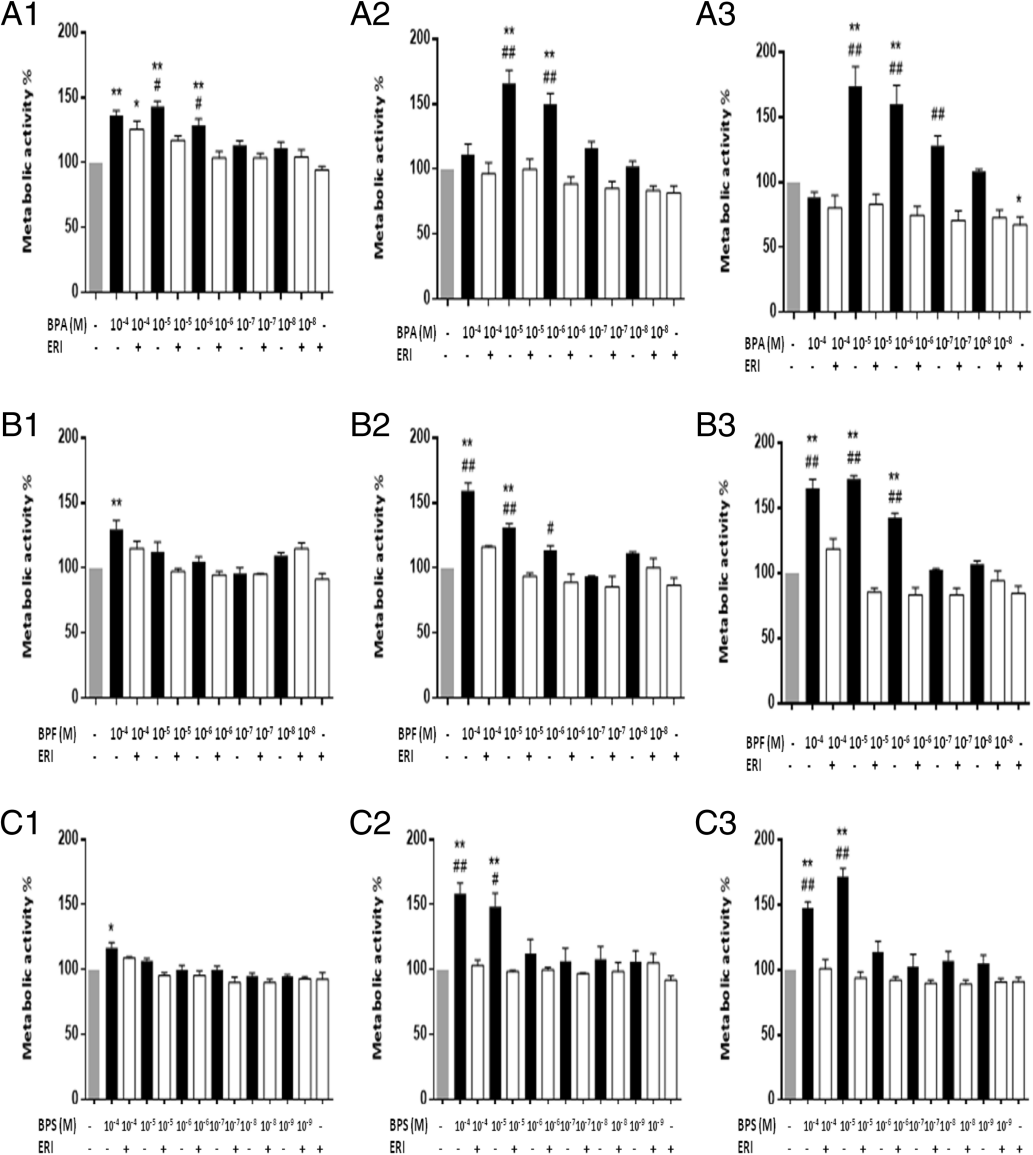
Metabolic activity of MCF-7 cells treated with BPA, BPF, and BPS ± ERI. Metabolic activity (MTT assay) of MCF-7 cells is shown following treatment with different doses of BPA, BPF, and BPS ± ERI for 24 h (**A1**, **B1**, **C1**), 48 h (**A2**, **B2**, **C2**), and 72 h (**A3**, **B3**, **C3**), respectively. Metabolic activity was calculated as percentage relative to control, and data are presented as mean + standard error of the mean (SEM) of at least three independent trials. Comparisons were performed between each treatment condition and control using analysis of variance (ANOVA) followed by Dunnett post hoc test (*p < 0.05 and **p < 0.001), and between the same treatment condition ± ERI using ANOVA followed by Tukey’s HSD (honestly significant difference) post hoc test (^#^*p* < 0.05 and ^##^*p* < 0.001)

**Fig. 2:**
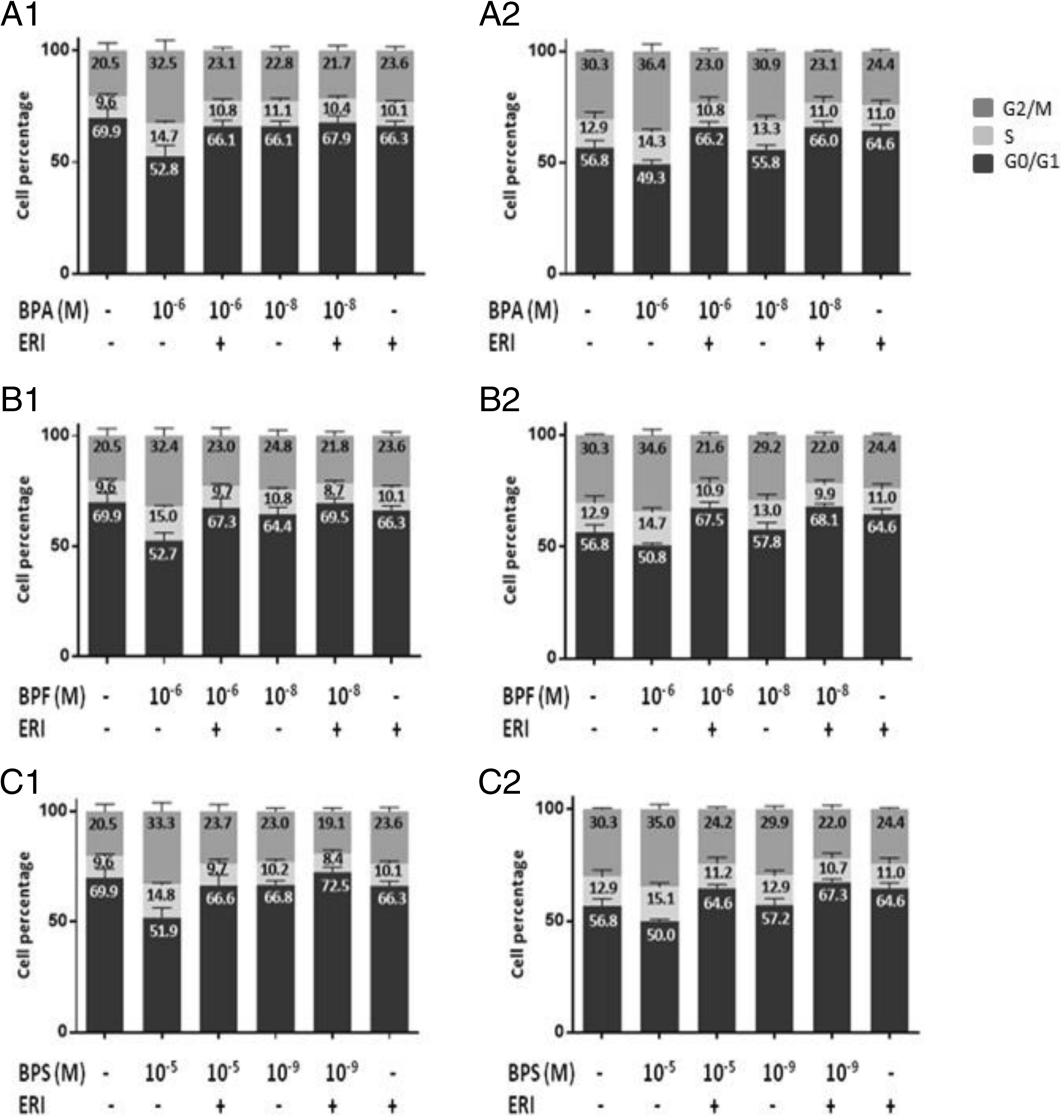
Cell cycle phase distribution of MCF-7 cells treated with BPA, BPF, and BPS ± ERI. Flow cytometry data depicted as mean percentage (+ SEM) of MCF-7 cells in different cell cycle phases is shown following treatment with exposure and functional doses of BPA, BPF, and BPS ± ERI for 24 h (**A1**, **B1**, **C1**) and 48 h (**A2**, **B2**, **C2**), respectively. Numbers in bars represent mean cell percentage within the corresponding cell cycle phase

**Fig. 3:**
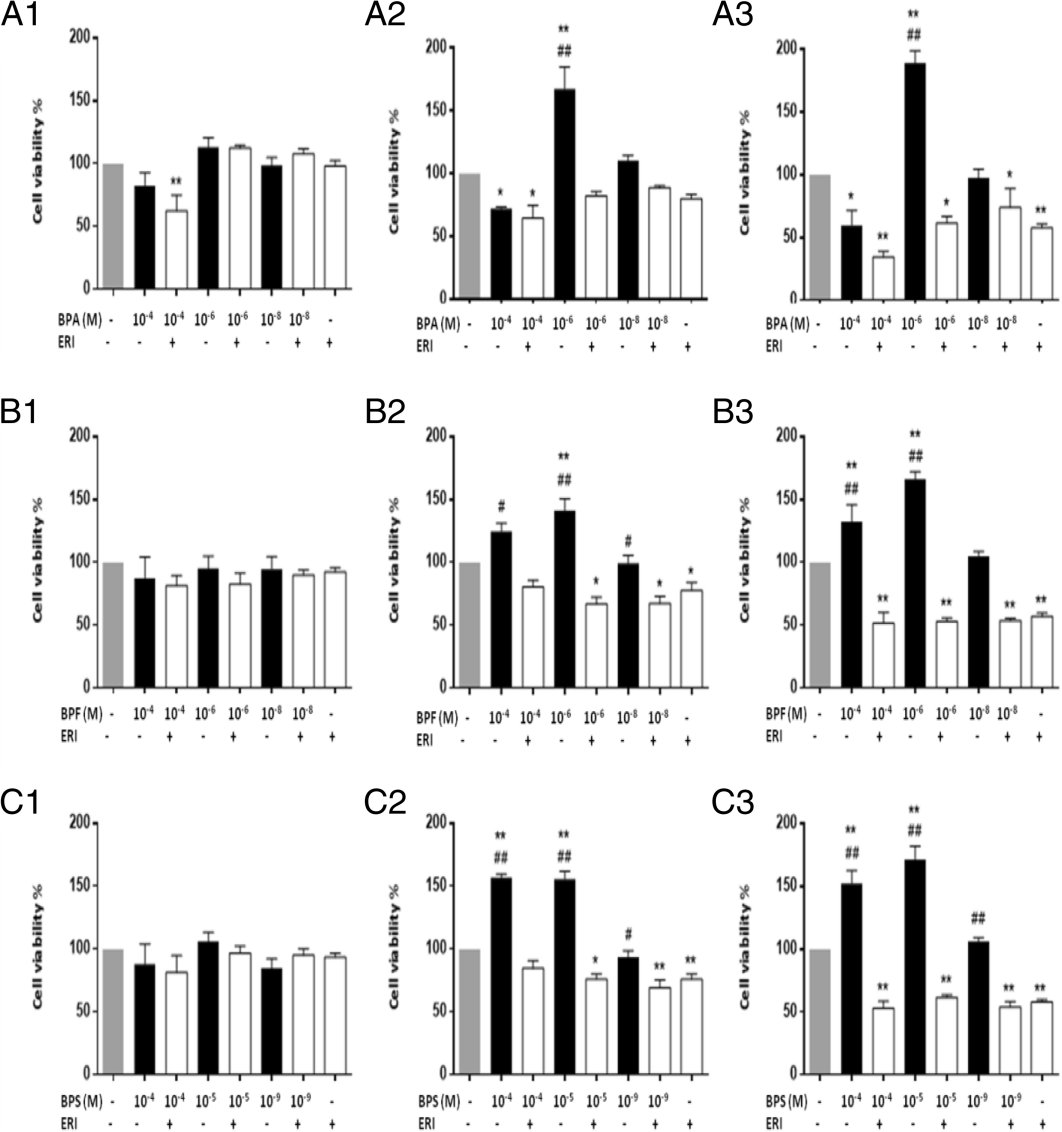
Cell viability of MCF-7 cells treated with BPA, BPF, and BPS ± ERI. Cell viability (trypan blue assay) of MCF-7 cells is shown following treatment with different doses of BPA, BPF, and BPS ± ERI for 24 h (**A1**, **B1**, **C1**), 48 h (**A2**, **B2**, **C2**), and 72 h (**A3**, **B3**, **C3**), respectively. Cell viability was calculated as percentage relative to control, and data are presented as mean + standard error of the mean (SEM) of at least three independent trials. Comparisons were performed between each treatment condition and control using analysis of variance (ANOVA) followed by Dunnett post hoc test (*p < 0.05 and **p < 0.001) and between the same treatment condition ± ERI using ANOVA followed by Tukey’s HSD (honestly significant difference) post hoc test (^#^*p* < 0.05 and ^##^*p* < 0.001)

**Fig. 4.**
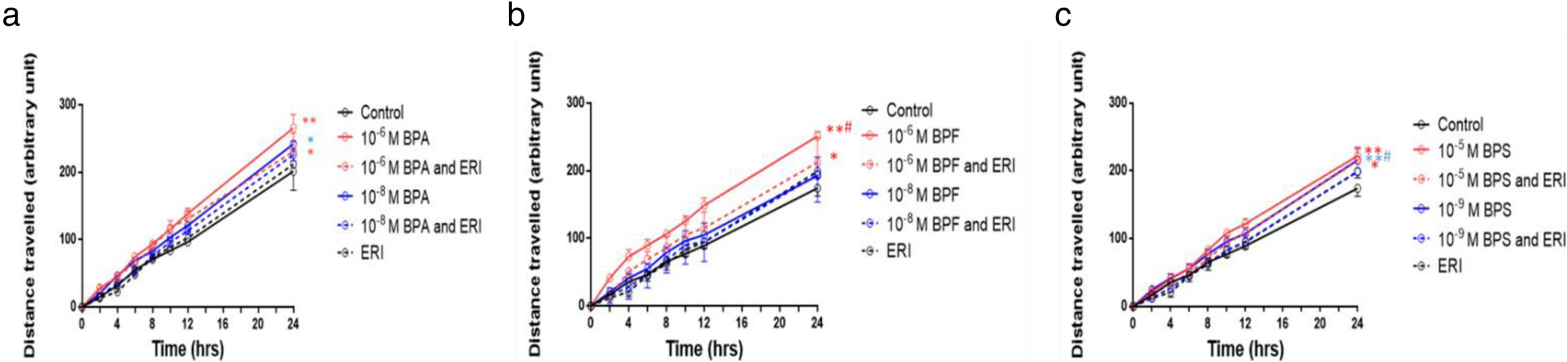
Cell migration assay of MCF-7 cells treated with BPA, BPF, and BPS ± ERI. Migration (scratch assay) of MCF-7 cells is shown following treatment with exposure and functional doses of BPA (**a**), BPF (**b**), and BPS (**c**) ± ERI. Data are presented as mean distance traveled (arbitrary unit) ± standard error of the mean (SEM) at time points 0, 2, 4, 6, 8, 10, 12, and 24 h in at least three independent trials. Comparisons were performed at every time point between each treatment condition and control using two-way analysis of variance (ANOVA) followed by Dunnett post hoc test (*p < 0.05 and **p < 0.001), and between the same treatment condition in the presence and absence of ERI using two-way ANOVA followed by Tukey’s HSD (honestly significant difference) post hoc test (^#^*p* < 0.05)

We observed a time- and dose-dependent increase in cell metabolic activity of MCF-7 cells after treatment with BPA, BPF, or BPS for 24, 48, and 72 h that was completely abolished in the presence of ERI at 48 and 72 h (Fig. 1). The lowest doses associated with a statistically significant increase in metabolic activity after 48–72 h of treatment were 10^−6^ M for BPA and BPF and 10^−5^ M for BPS, and they induced an increase in metabolic activity by 50 to 100% relative to control at 72 h (Fig. 1). These doses were considered “equipotent” and used in the remaining assays, together with the human exposure doses. Consistently, the three bisphenols resulted in dose- and ER-dependent increases in both S and G2/M proportions of the cell cycle at 24 h of treatment in MCF-7 cells (Fig. 2), followed by a significant increase in percentage cell viability at 48 and 72 h of treatment (Fig. 3) (Fig. 2 and Additional file 1: Figure S4). This, and the fact that trypan blue and cell cycle results did not show marked changes in cell death, supports the expectation that the increased S-G2/M proportion of treated cells represents increased cell proliferation rather than cell cycle arrest. Both the functional and exposure doses of BPA, BPF and BPS increased cell migration in a time- and dose-dependent manner, with the distance traveled being statistically significant in all conditions except with the exposure dose of BPF (Fig. 4 and Additional file 1: Figure S5). Addition of ERI abolished this increase in the exposure doses and partially prevented it in the functional doses of BPA, BPF, and BPS (Fig. 4). The increase in cell proliferation and migration with the three bisphenols was not associated with any morphological changes in MCF-7 cells (Additional file 1: Figure S6).

There was no increase in the metabolic activity, viability, and migration of MDA-MB-231 cells after treatment with the three bisphenols, hence, confirming the role of ER in mediating the effects of exposure to endocrine disruptors on cell proliferation and migration. Of note, a modest (< 50%) decrease (rather than increase) in cell viability was observed only at late time points and very high doses (10^−4^ M) (Additional file 1: Figures S7, S8, and S9).

Since the three bisphenols resulted in significant changes in proliferation and migration of MCF-7 cells and not of MDA-MB-231 at 48 and 72 h, we assessed the effects of these EDCs on molecular effects in MCF-7 cells only. We also focused on early time points (24 and 48 h), coinciding with molecular readouts that likely precede (hence, are causal to rather than resultant from) the observed phenotypic manifestation of cancer progression.

### Bisphenols induced an ER-dependent increase in *DNMT1* RNA expression and differential increase in *TETs 2* and *3* RNA expression but did not change their overall enzymatic activities

There were modest increases (< 2 folds) in *DNMT1* RNA expression in MCF-7 cells after 24 h of treatment with the functional doses of BPA, BPF, and BPS and with the human exposure dose of BPA (except for a 2.5-fold increase with the BPS functional dose), and these effects were inhibited by ERI. RNA expression levels of *DNMT3a* and *DNMT3b* and overall DNMT enzymatic activity were not altered with the three bisphenols (Additional file 1: Figure S10A).

Similarly, there were ER-dependent modest increases (< 2 folds) in *TET2* RNA expression after 24 h of treatment with the functional and exposure dose of BPA and BPS and similar increases in *TET3* RNA expression after 24 h of treatment with the functional and exposure dose of BPA. RNA expression levels of *TET1* and overall TET enzymatic activity were not altered with the three bisphenols (Additional file 1: Figure S10B).

### Bisphenols showed a trend of *LINE-1* hypomethylation that was ER-dependent with BPA and BPF

We tested the effects of functional (equipotent) doses of BPA and its analogs on *LINE-1* methylation, which additionally served as a screening tool to determine the earliest time points and concentrations at which overall methylation alterations occur, so that these conditions can be subsequently analyzed in-depth with genome-wide methylation profiling. BPA and BPF (and to a lesser extent BPS) were associated with a trend towards decreased *LINE-1* methylation in MCF-7 cells at 48 h, which was not observed at 24 h. At 48 h, mean decrease in *LINE-1* methylation was 1.63% and 2.14% with BPA and BPF, respectively. Of note, the hypomethylation of *LINE-1* at 48 h was completely abolished in the presence of ERI (Additional file 1: Figure S11)**.**

Since there were more changes in *LINE-1* methylation after treatment of MCF-7 cells with functional doses for 48 h compared to 24 h, the subsequent methylome-wide association experiment was performed at 48 h.

### Bisphenols induce differential DNA methylation alterations in several CpG sites and regions located mostly in gene promoters and exons

We used two approaches for the epigenome-wide analysis leading to the identification of differentially methylated CpG probes (DMPs) and differentially methylated regions of CpG clusters (DMRs). Both approaches showed that functional doses of each of BPA, BPF, and BPS have a profound impact on the DNA methylome, leading to a large number of statistically significant DMPs or DMRs compared to their corresponding controls (Additional file 2: Table S3), even when using stringent correction (SVA) for batch and confounder effects. Lambda values and q-q plots were not indicative of major inflation (Additional file 1: Figure S12).

**Fig. 5:**
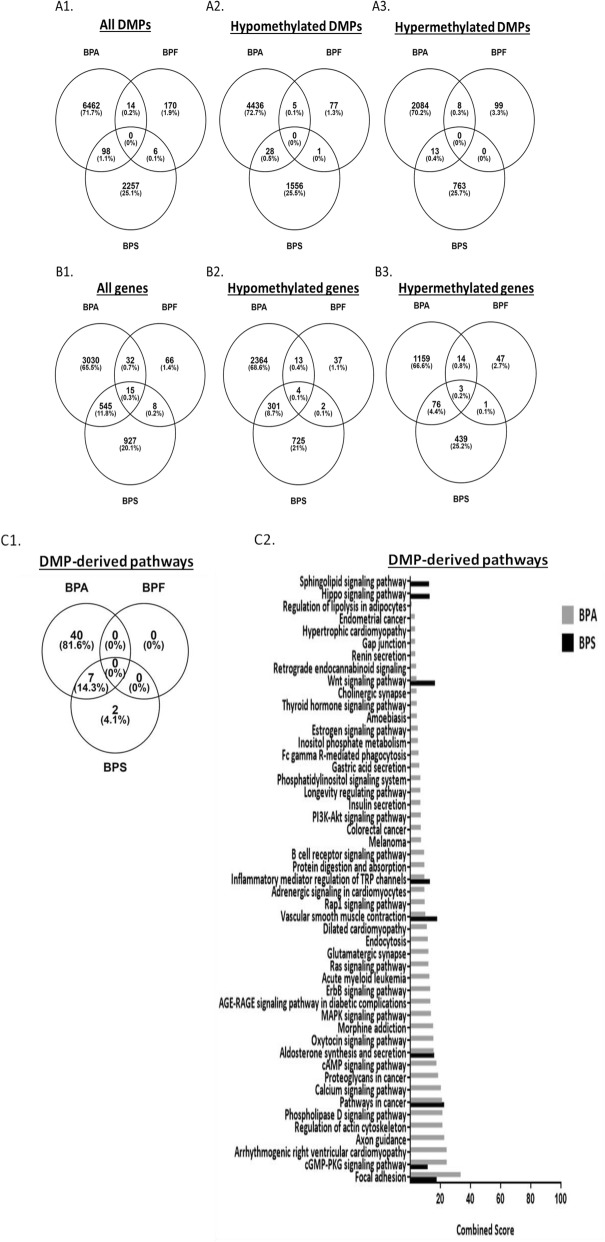
DNA methylome-wide analysis of DMPs in MCF-7 cells treated with BPA, BPF, and BPS. Differentially methylated probes (DMPs) were analyzed in MCF-7 cells treated for 48 h with exposure and functional doses of BPA, BPF, and BPS treatment conditions when compared to control. Venn diagrams of DMPs and genes encompassing DMPs of BPA, BPF, or BPS are shown in **A1**–**A3** and **B1**–**B3**, respectively. Pathways of genes encompassing DMPs of BPA, BPF, and BPS were detected based on KEGG pathway database using Enrichr (http://amp.pharm.mssm.edu/Enrichr/) and shown in **C1** and **C2**. BPF was not included in **C2**, because its DMP-derived genes were not significantly involved in any pathway (as shown in **C1**)

Although BPA, BPF, and BPS share similar chemical structures (Additional file 1: Figure S1), they exhibited differential effects on the methylome at equipotent functional doses. In particular, both DMP and DMR analyses showed that the effect was consistently strongest for BPA and weakest for BPF (Additional file 2: Table S3) and that the majority of identified DMPs, DMRs, or genes encompassing the DMPs/DMRs did not overlap between any of the three compounds (Fig. 5 and Additional file 1: Figure S13). A larger proportion of the DMPs, DMRs, or differentially methylated genes was hypomethylated in BPA and BPS treatments, while similar proportions of hypo- and hyper-methylation were observed with BPF. The significant DMPs, DMRs, and their corresponding genes are listed in Additional file 3. Even though BPA, BPF, and BPS showed differential epigenetic effects at the gene level, there were similarities observed among the compounds at a more global genomic level. Specifically, the genomic distribution of the DMPs and DMRs were strikingly similar among the three bisphenols, showing enrichment in promoter regions and exons and diminishment in intergenic regions, compared to random probes (Additional file 1: Figure S14). To note, the three CpG sites validated with pyrosequencing spanned low to high ranges of methylation values and showed a significant correlation (*P* = 9.97 × 10^−10^) between the array and sequencing results (Additional file 1: Figure S15).

### Differentially methylated genes of BPA and BPS are involved in cancer-related pathways

Pathway analysis with KEGG database based on the DMP-derived genes showed 47 pathways for BPA, nine for BPS, and none for BPF. Seven out of nine pathways of BPS were common with those of BPA, with focal adhesion, cyclic guanosine monophosphate (cGMP)-protein kinase G (PKG) signaling, and cancer pathways having the highest combined score (Fig. 5C2). Additionally, several subsets of cancer pathways were observed with BPA-induced DMPs (proteoglycans in cancer, acute myeloid leukemia, melanoma, colorectal cancer, and endometrial cancer). Moreover, the estrogen signaling pathway was detected with BPA-induced DMPs. As for the DMR-derived genes, using the KEGG database revealed 32 significant pathways for BPA but none for BPF and BPS, so comparison of pathways among the three compounds through this approach was not possible. However, comparing the pathways of genes obtained with DMPs to those obtained with DMRs for BPA revealed 21 out of 32 common pathways, with glutamatergic synaptic, calcium signaling, cGMP-PKG signaling, and phospholipase D pathways having the highest combined score. Interestingly, Wnt signaling pathway was common between the pathways derived from genes encompassing DMPs and DMRs induced by BPA (Additional file 1: Figure S13).

### Most bisphenol-induced differentially methylated sites and regions were ER-dependent

**Fig. 6:**
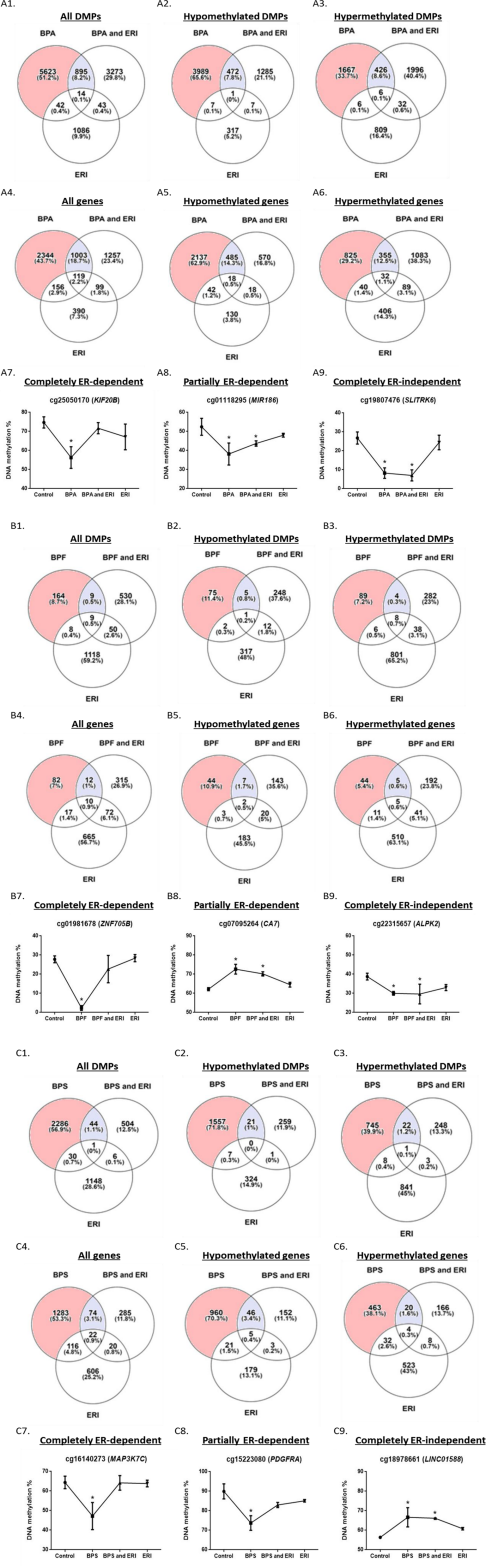
DNA methylome-wide analysis of DMPs in MCF-7 cells treated with BPA, BPF, and BPS ± ERI. Differentially methylated probes (DMPs) were analyzed in MCF-7 cells treated for 48 h with functional doses of BPA, BPF, and BPS with or without ERI and ERI alone when compared to control. Venn diagrams of DMPs and genes encompassing DMPs of BPA ± ERI, BPF ± ERI, and BPS ± ERI are shown in **A1**–**A6**, **B1**–**B6**, and **C1**–**C6**, respectively. Representative figures of DMPs (*genes*) altered by BPA, BPF, or BPS treatment conditions and being dependent on (**A7**, **B7**, **C7**), partially dependent on (**A8**, **B8**, **C8**), or completely independent of (**A9**, **B9**, **C9**) ERI, respectively. Data are presented as mean ± standard deviation (SD) of two independent trials. Comparisons were performed between each treatment condition and corresponding control using robust linear model (RLM) (*p value < 0.05)

**Fig. 7:**
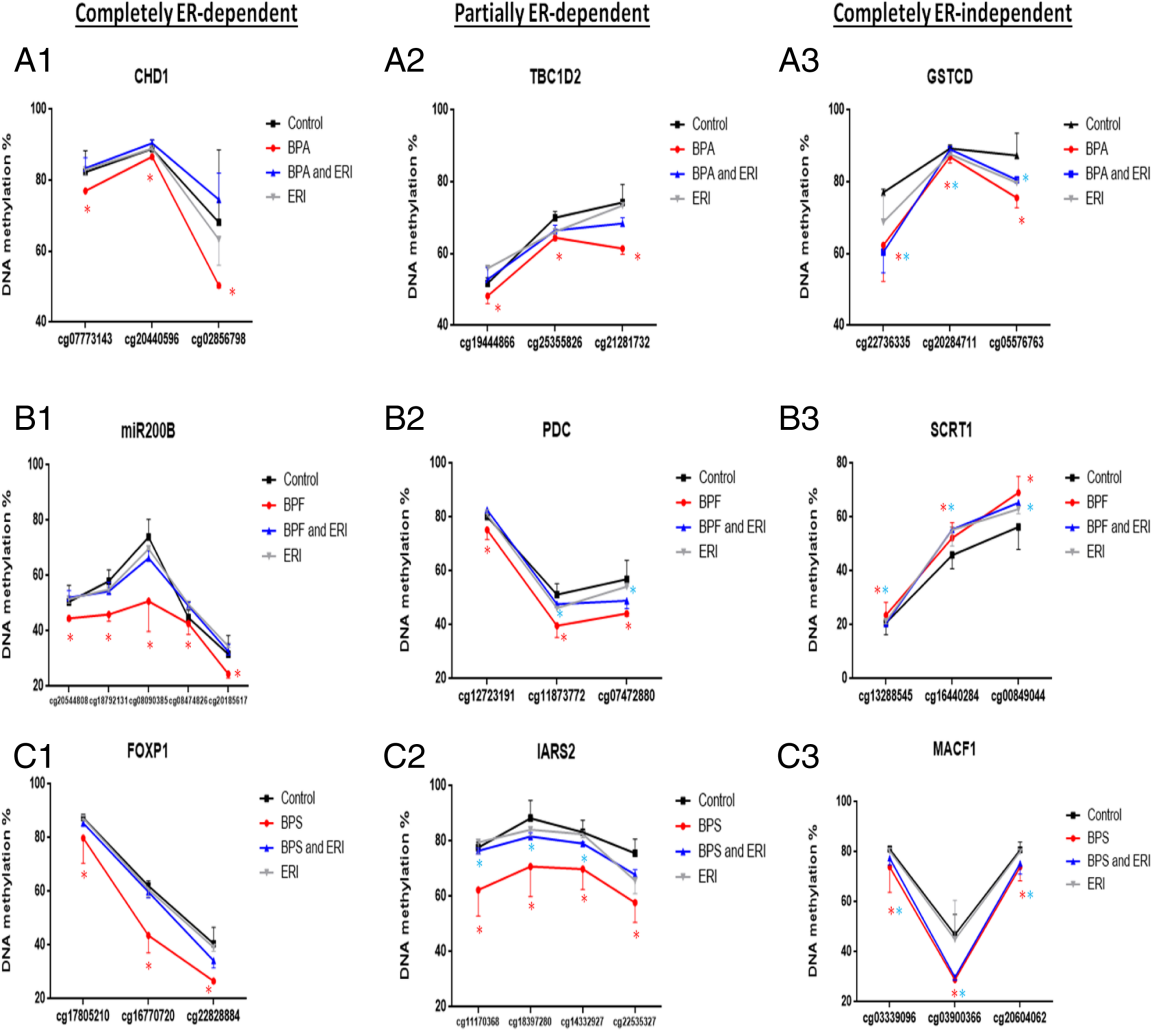
Representative figures of DMRs in MCF-7 treated with BPA, BPF, and BPS ± ERI. Representative figures of differentially methylated regions (DMRs) in MCF-7 treated for 48 h with functional doses of BPA, BPF, and BPS and being completely dependent on (**A1**, **B1**, **C1**), partially dependent on (**A2**, **B2**, **C2**), or completely independent of (**A3**, **B3**, **C3**) ERI, respectively. Data are presented as mean ± standard deviation (SD) of two independent trials. Comparisons were performed between each treatment condition and control using robust linear model (RLM) (*p value < 0.05)

Most of the revealed DMPs, DMRs, and genes encompassing them were not associated with the bisphenols’ treatments when combined with ERI and were hence dependent on ER (Fig. 6 and Additional file 1: Figure S16). Figures 6 and 7 show representative DMPs and DMRs (with the most significant and highest effect sizes) which were completely dependent on, partially dependent on, or completely independent of ER. Interestingly, 25 out of 47 pathways pertaining to DMP-derived genes of BPA were ER-dependent, including cAMP, MAPK, estrogen, and Wnt signaling pathways. Moreover, all of the pathways pertaining to DMP-derived genes of BPS were ER-dependent (data not shown).

### Some differentially methylated genes and cancer-related pathways induced by BPA and BPS were also dysregulated in ER-positive breast cancer patients

**Fig. 8:**
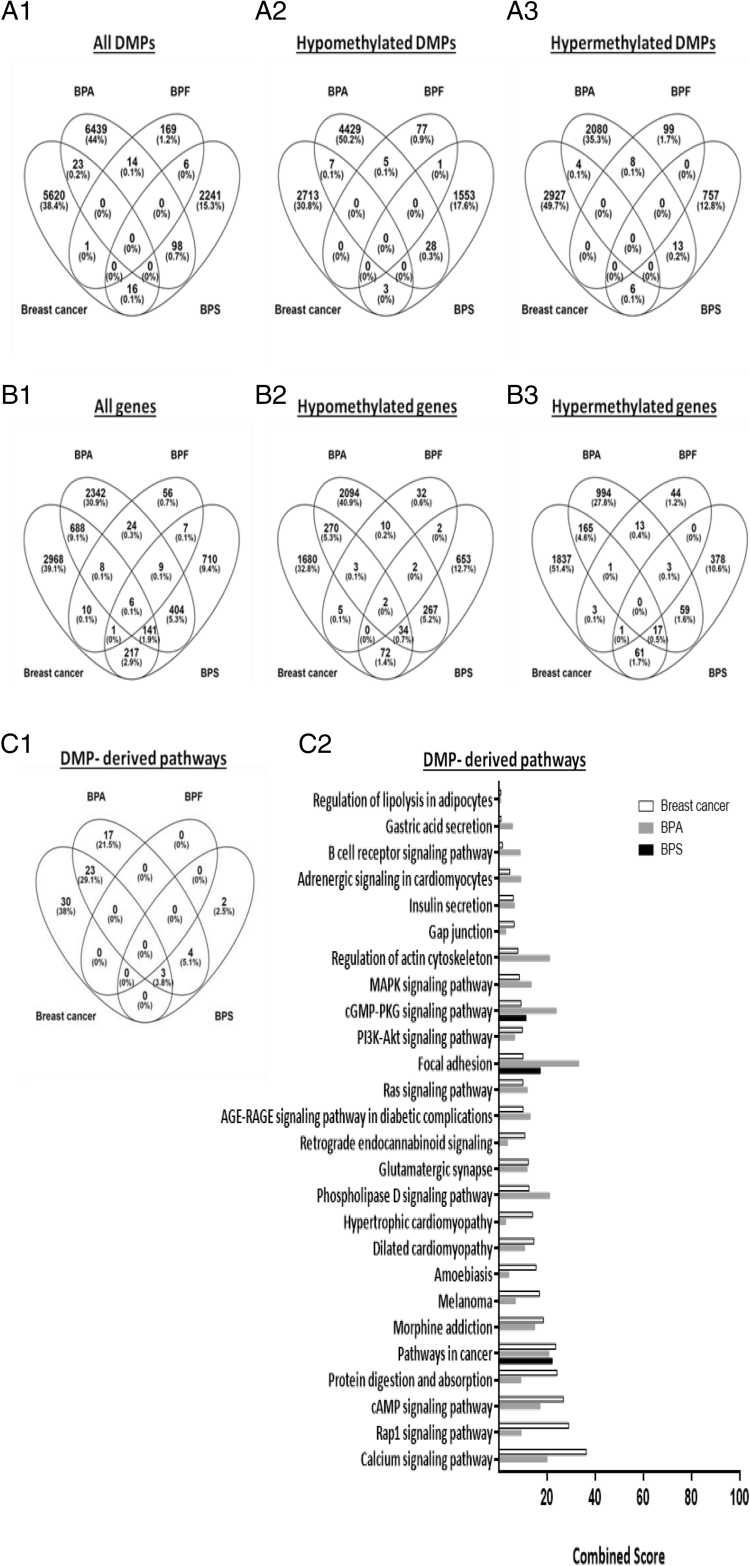
Comparison of BPA-, BPF-, and BPS-induced DMPs in MCF-7 cells with DMPs in breast cancer patients. DMPs and genes encompassing DMPs in MCF-7 cells treated for 48 h with functional doses of BPA, BPF, and BPS were compared with those of 595 ER-positive tumor tissues relative to 124 normal-adjacent tissues of The Cancer Genome Atlas (TCGA) breast cancer patients. Venn diagrams of DMPs and genes encompassing DMPs of BPA, BPF, BPS, and breast cancer are shown in **A1**–**A3** and **B1**–**B3**, respectively. Pathways of the genes encompassing DMPs of BPA, BPF, BPS, and breast cancer were detected based on KEGG pathway database using Enrichr (http://amp.pharm.mssm.edu/Enrichr/) and shown in **C1** and **C2**. BPF was not included in **C2**, because its DMP-derived genes were not significantly involved in any pathway (as shown in **C1**)

As shown in Additional file 3, the methylation percentages of 5660 CpGs were statistically significantly different between ER-positive tumor and normal tissues. Aberrations in DNA methylation of 11 CpG sites (7 hypomethylated and 4 hypermethylated) were common with BPA and of 9 CpG sites (6 hypermethylated and 3 hypomethylated) were common with BPS (Fig. 8 and Additional file 2: Table S4). There were no similarly dysregulated CpGs between BPF (having the least number of significant DMPs and DMRs) and breast cancer tissues. Of note, there were 309 common hypomethylated genes and 183 common hypermethylated genes with BPA, 108 common hypomethylated genes and 79 common hypermethylated genes with BPS, and only 10 common hypomethylated genes and 5 common hypermethylated genes with BPF. Interestingly, pathway analysis revealed the majority (26/47) of the BPA pathways to be common with those of breast cancer, with pathways in cancer, focal adhesion, and cGMP-PKG signaling pathways being common between BPA, BPS, and breast cancer (Fig. 8).

## Discussion

In this study, we showed that BPA and its analogs induced ER-dependent increases in cell proliferation, migration, and S-G2/M cycling proportions in MCF-7 cells. The functional and exposure doses at which these effects were observed were identical between BPA and BPF but one order of magnitude higher for BPS, consistent with previous studies showing that the estrogenic activity of BPS is around 10 times less than BPA and BPF [20, 21]. While only few in vitro studies showed that BPF [36, 37] and BPS [19] increase the proliferation of breast cancer cell lines (MCF-7 or MCF-7 CV), several showed that BPA increases the proliferation of these same cell lines [4, 5, 9, 36–41] and few investigated whether this increase is ER-dependent [19, 40]. We also observed that the functional dose of the three bisphenols increased the percentage of cells in the G2/M and S phases in MCF-7 cells after 24 h, and this was also shown in the literature for BPA and BPF [36]. Similar to our results, BPA increased the cell migration and metastasis of breast cancer cell lines [19, 42]. However, in the literature, only one study tested the effects of BPF and BPS on cell migration of breast cancer cells and showed that, similarly to BPA, BPF and BPS increased cell migration and altered the expression of epithelial to mesenchymal transition markers in MCF-7 CV cells [19]. As expected, we did not observe any change in the cell metabolic activity, viability, and migration of ER-negative MDA-MB-231 cells consistently with other studies which observed no effects of BPA on the proliferation of these cells [37, 41].

In this study, the functional doses of the three bisphenols were associated with a trend towards *LINE-1* hypomethylation in MCF-7 cells. However, taking into account that *LINE-1* is a repetitive sequence that comprises around 17% of the genome [43], a decrease of only 1% in *LINE-1* methylation is considered biologically relevant. Global DNA hypomethylation has been associated with cancer development [44] including breast cancer [18, 45]. In the literature, global DNA hypomethylation was reported in MCF-7 cells after 48 h of treatment with 10^−7^ M and 10^−6^ M BPA using 5-mC Elisa kit, but no changes were reported in the same cell line treated for 5 weeks with 10^−5^ M and 10^−6^ M BPA using the “gold standard” HPLC-MS assay [14, 46]. Discrepancies in results might be attributed to different treatment duration or measurement methods. Besides, a recent study showed hypermethylation in two transposons (*MaLR* and *Mariner-2*) out of eight analyzed in MCF-7 cells treated with 10^−6^ M BPS for 24 h [47]. However, no study measured *LINE-1* methylation in breast cell lines treated with any of BPA, BPF, or BPS.

Genome-wide DNA methylation revealed that the three bisphenols significantly altered the DNA methylation of several CpG sites and CpG regions that were located mostly in promoters and exons, yet they showed minimal degree of overlap. In both DMP and DMR analyses, BPA was associated with the largest number of DMPs and DMRs followed by BPS then BPF; hence, BPA had the strongest effect on the DNA methylome among the three bisphenols. Noteworthy, the majority of DMPs and DMRs altered by bisphenols were ER-dependent. In the literature, only one methylome-wide analysis was performed with MCF-7 cells treated for 5 weeks with 10^−5^ M and 10^−6^ M BPA using an older generation Infinium Human Methylation450 BeadChip arrays, and showed that both doses of BPA induce hypermethylation in tumor suppressor genes and hypomethylation in oncogenes [14]. Out of the reported 32 hypomethylated genes and 45 hypermethylated genes with both doses of BPA, three genes were similarly hypomethylated (*ZNF423*, *SYT4*, *IMMP2L*) and five genes were similarly hypermethylated (*THSD4*, *SETBP1*, *NTM*, *HLA-DRB1*, *AFF1*) with our 48-h treatment with BPA. No epigenome-wide study has so far been reported for BPF and BPS in breast cells.

The potential effect of the three bisphenols on the expression of DNA methylation enzymes in breast cells has not been previously addressed in the literature. However, BPA showed tissue-specific alterations in *DNMT* gene expression in several tissue types other than the breast [48, 49]. In this study, we showed that although the three bisphenols induced ER-dependent increase in *DNMT1* gene expression and showed differential ER-dependent effects on *TET* (*2* and *3*) gene expression levels, these alterations barely exceeded the commonly used twofold change limit and were not translated into alterations in enzymatic activity. Hence, we propose that the DNA methylation aberrations induced by the three bisphenols in MCF-7 cells were potentially mediated by changes in signaling pathways (including the ER) without directly affecting enzymatic activities of DNMTs/TETs.

Pathway analysis revealed that the genes encompassing differentially methylated probes by BPA and BPS were involved in seven common pathways whereby focal adhesion, cGMP-PKG signaling, and cancer pathways had the highest score. However, those of BPF were not statistically significantly involved in any pathway. All of the pathways dysregulated by BPS were ER-dependent, yet roughly half of the pathways dysregulated by BPA were ER-dependent including cAMP, MAPK, estrogen, and Wnt signaling pathways. Of note, two other tools (Go Biological and WikiPathways) showed that genes differentially methylated by BPA and BPS were involved in Wnt signaling cascade (data not shown), a pathway that was also obtained by the KEGG database for DMRs of BPA. This pathway controls development and stemness and has been tightly linked to cancer development and metastasis [50].

When comparing the methylation data with those of clinical data, the overlap in the genes encompassing the DMPs was the highest with BPA and the least with BPF. As a matter of fact, pathway analysis using KEGG revealed that the discovered top three common pathways between BPA and BPS with the highest combined score, namely focal adhesion, cGMP - PKG signaling, and cancer pathways, were also common with breast cancer.

### Limitations

This study is limited by several factors related to the choice of cells and treatment duration. The main limitation is that it was only performed on breast cancer cell lines, so similar assays on normal-like breast epithelial cells or stem cells are warranted to determine the carcinogenic potential and mechanisms of the BPA analogs in comparison with BPA. Besides, a pure control without vehicle was not included in most of the assays. Knowing that DNA methylation is not stable upon cell culture, a pure control could have been particularly useful in the DNA methylome-wide assay to exclude any DNA methylation changes related to the solvent. However, assuming that changes induced by the solvent will occur in both bisphenol- and solvent-treated cells and because of limitations in resources, we included only solvent-treated cells as controls. Moreover, although we exposed the MCF-7 cells to human exposure doses of BPA, BPF, and BPS, some of the molecular changes and epigenetic aberrations were observed at larger doses. However, treatment duration was only 2 days compared with a lifetime exposure to BPA, BPF, and BPS, so higher doses may be important to investigate the mechanisms of the three bisphenols in this short treatment period. Additionally, the levels of BPA, BPF, and BPS vary between different age groups and different human compartments; for instance, BPA levels were higher in children than in adults and concentrated in human placental and fetal liver tissues [51, 52]. It is essential to take into consideration that many papers reported a high occupational BPA exposure, whereby BPA manufacturers had urinary and semen BPA levels approaching the functional dose used in this study and sometimes even higher [22]. Notably, in this study, we fulfilled several of the recommended guidelines concluded in a recent review on epigenetics of BPA in regard of use of multiple doses, multiple time-points, and integration of epigenetic data with other molecular and phenotypic readouts [12].

## Conclusions

Despite these limitations, this is the first study to elucidate the epigenetic-linked mechanisms of BPF and BPS in human carcinoma cells and compare them to those of BPA. Similarly to other studies, we showed an increase in ER-dependent cancer cell proliferation and migration with the three bisphenols in breast cancer cells, with BPS being 10 times less potent than BPA and BPF in its cell proliferation effects. However, at equipotent doses, the three bisphenols showed differential DNA methylation alterations which were likely not mediated by effects on DNA (de)methylation enzymes. BPA had the strongest effect on the DNA methylome, followed by BPS then BPF, and the majority of bisphenol-induced DNA methylation alterations were dependent on ER pathway. Differentially methylated genes by BPA and BPS were involved in focal adhesion, cGMP-PKG, and cancer pathways (including several cancer pathway subsets), which were also dysregulated in ER-positive breast cancer tumor tissues. DNA methylation aberrations induced by BPA and BPS were also involved in Wnt signaling that is positively linked to cancer progression. We conclude that the three bisphenols have important epigenetic effects in breast cell lines, with those of BPA and BPS overlapping with cancer-related pathways in clinical breast cancer models, hence, warranting further investigation regarding the safety of BPA and its derivatives.

## Additional files

Additional file 1: Supporting Figures S1–S16. (DOCX 8.22 kb)

Additional file 2: Supporting Tables S1–S4. (DOCX 24.9 kb)

Additional file 3: DNA methylome-wide results in biphenol-treated MCF-7 cells and in TCGA breast cancer patients. DNA methylome-wide results (DMPs and DMRs) of MCF-7 cells treated with BPA, BPF and BPS ± ERI for 48 hrs and DNA methylome-wide results (DMPs) of ER-positive breast cancer tissues when compared to adjacent normal tissues in TCGA breast cancer patients. (XLSX 2.310 kb)

## Data Availability

All data generated or analyzed during this study are included in this published article [and its supplementary information files].

## Abbreviations

ANOVA: Analysis of variance
ATCC: American Type Culture Collection
BPA: Bisphenol A
BPF: Bisphenol F
BPS: Bisphenol S
BSA: Bovine serum albumin
cGMP: Cyclic guanosine monophosphate
CHAPS: 3[(Cholamidopropyl)-dimethyl-ammonium]-1-propanesulfonate
DMEM: Dulbecco’s modified Eagle’s medium
DMP: Differentially methylated CpG probe
DMR: Differentially methylated region
DNMT: DNA methyltransferase
EDC: Endocrine disrupting compound
EDTA: Ethylenediaminetetraacetic acid
ER: Estrogen receptor
ERI: Estrogen receptor inhibitor
FBS: Fetal bovine serum
FDR: False discovery rate
HSD: Honestly significant difference
IARC: International Agency for Research on Cancer
NAC: No amplification control
NTC: No template control
OD: Optical density
PBS: Phosphate-buffered saline
PCA: Principal component analysis
PKG: Protein kinase G
RLM: Robust linear model
RT-PCR: Reverse transcription-polymerase chain reaction
SD: Standard deviation
SEM: Standard error of the mean
SVA: Surrogate variable analysis
TCGA: The Cancer Genome Atlas
TET: Ten-eleven translocation
*β*: Beta value
Δβ: Delta beta
