# Supplementary material for: DNA methylome-wide alterations associated with estrogen receptor-dependent effects of bisphenols in breast cancer
Source: Clin Epigenetics. 2019 Oct 10;11:138. doi: 10.1186/s13148-019-0725-y (PMC6785895; doi:10.1186/s13148-019-0725-y)

**Supporting Figure 1.** Chemical structures of bisphenol A (BPA), bisphenol F (BPF) and bisphenol S (BPS)

**
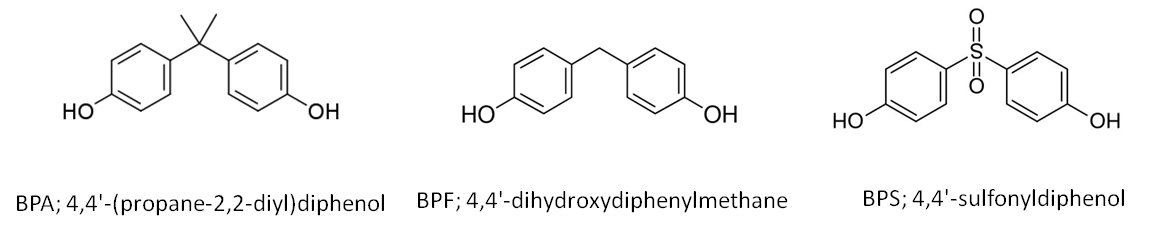
**

**Supporting Figure 2.** Density plots (A1, A2) and density bean plots (B1, B2) of beta values pertaining to different treatment conditions before and after FunNorm filtration, respectively


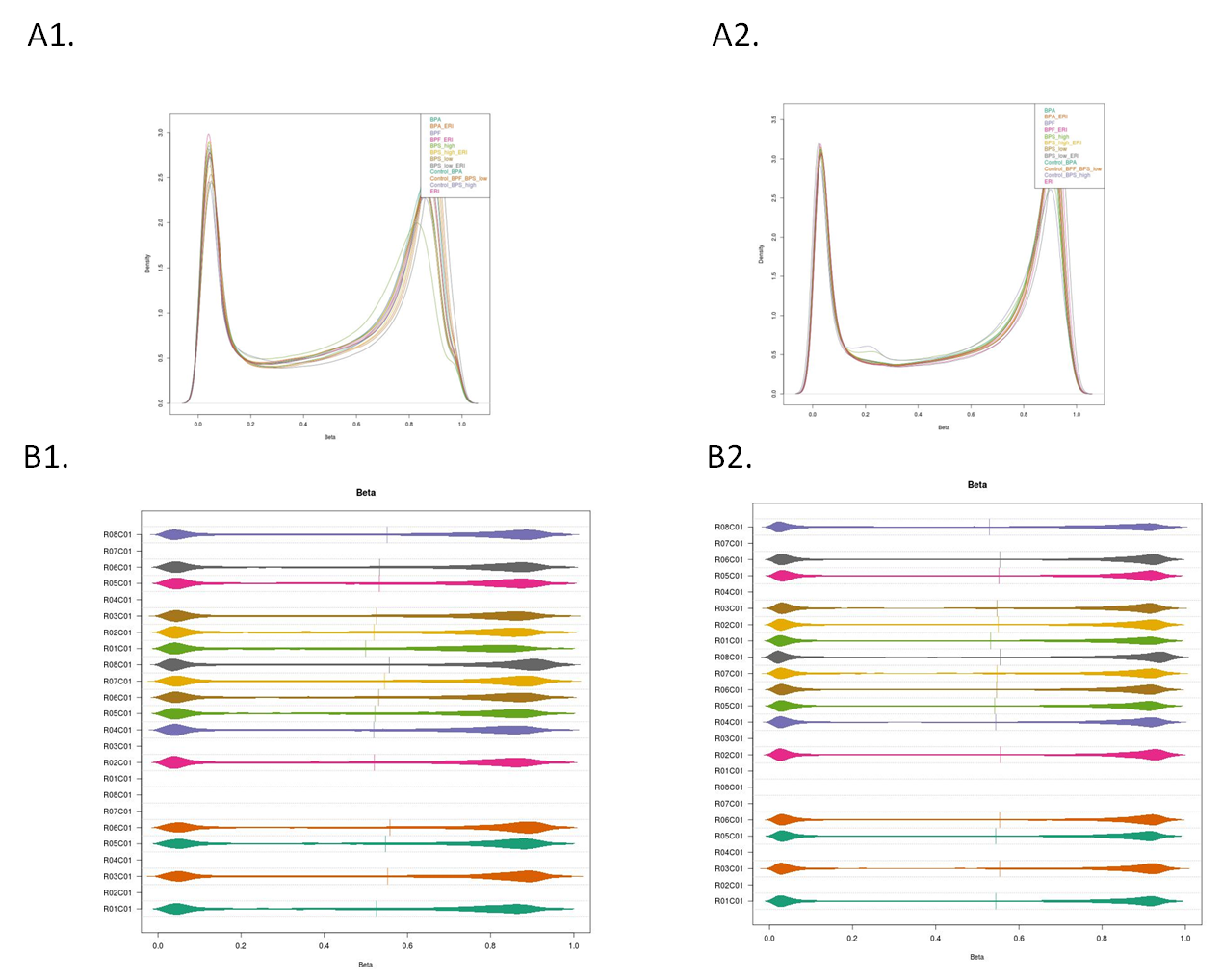


**Supporting Figure 3.** Principal component analysis (PCA) of the contribution of different variables before (A) and after (B) surrogate variable analysis (SVA) correction

1: treatment condition; 2: trial number; 3: sentrix ID; 4: sentrix position


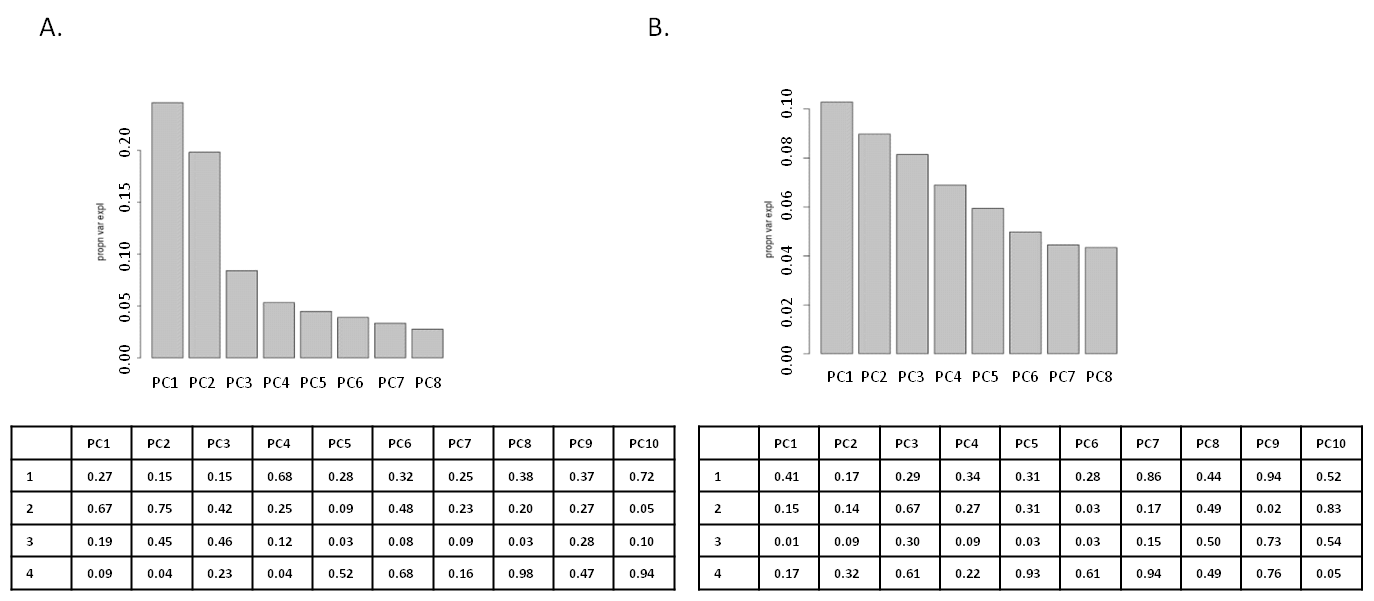


**Supporting Figure 4.** Flow cytometry figures of a representative trial depicting cell cycle phases of MCF-7 cells following treatment with exposure and functional doses of bisphenol A (BPA), bisphenol F (BPF) and bisphenol S (BPS) ± estrogen receptor inhibitor (ERI) for 24hrs


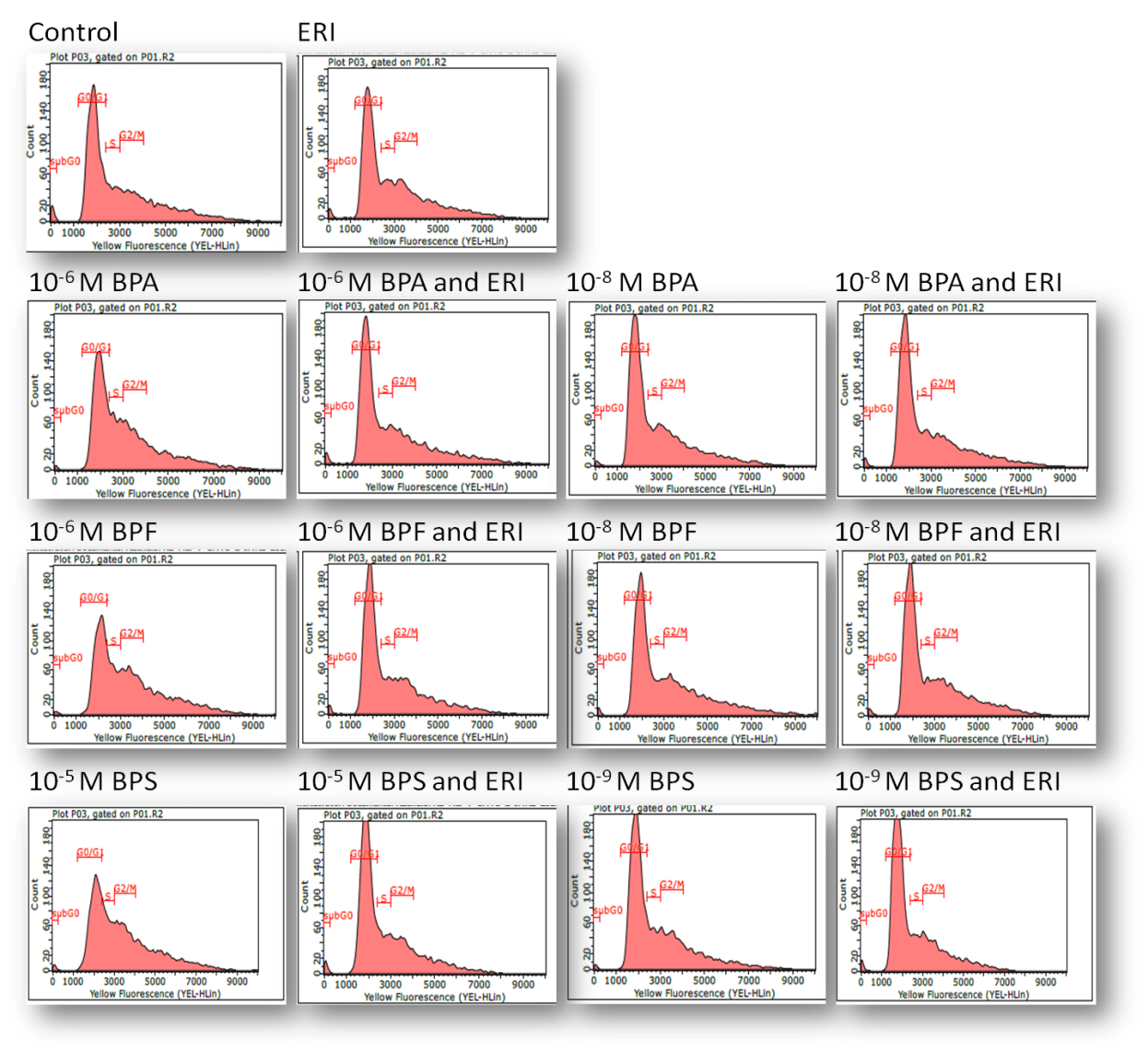


**Supporting Figure 5.** Representative figures of MCF-7 cell migration (scratch assay) showing MCF-7 cells treated with functional doses of bisphenol A (BPA) (A), bisphenol F (BPF) (B) and bisphenol S (BPS) (C) ± estrogen receptor inhibitor (ERI) at time points 0, 12, 24 hrs

Figures were captured using light microscope (5× magnification), and results of treatment conditions that were significantly different from control are highlighted in green.


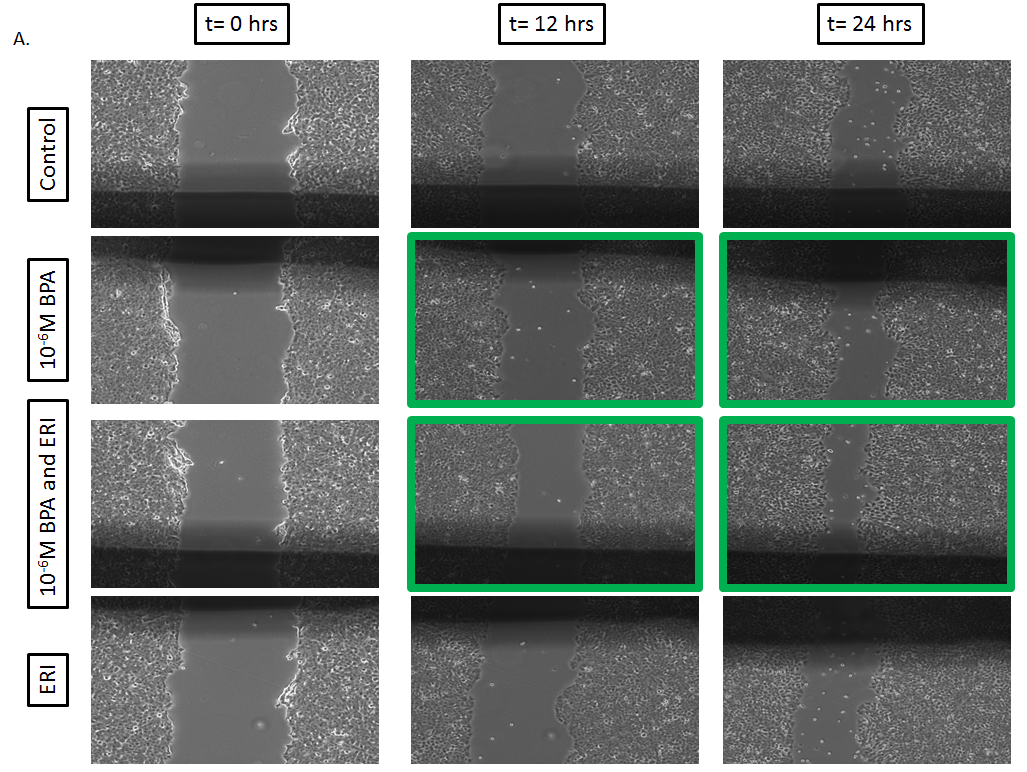


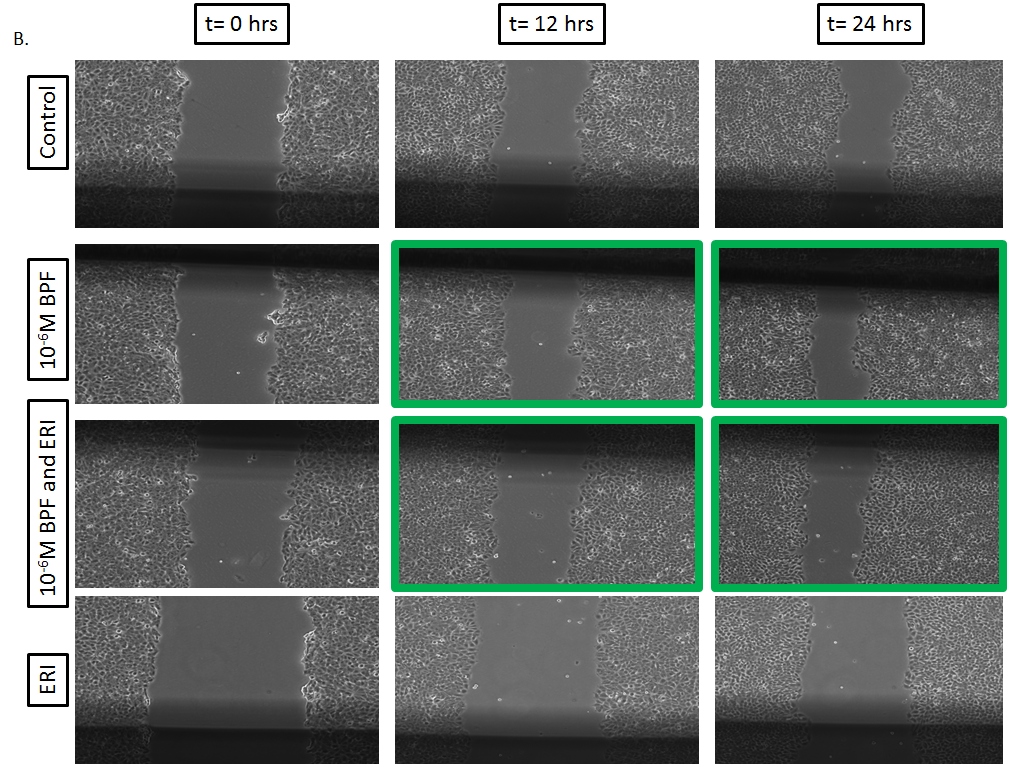


**
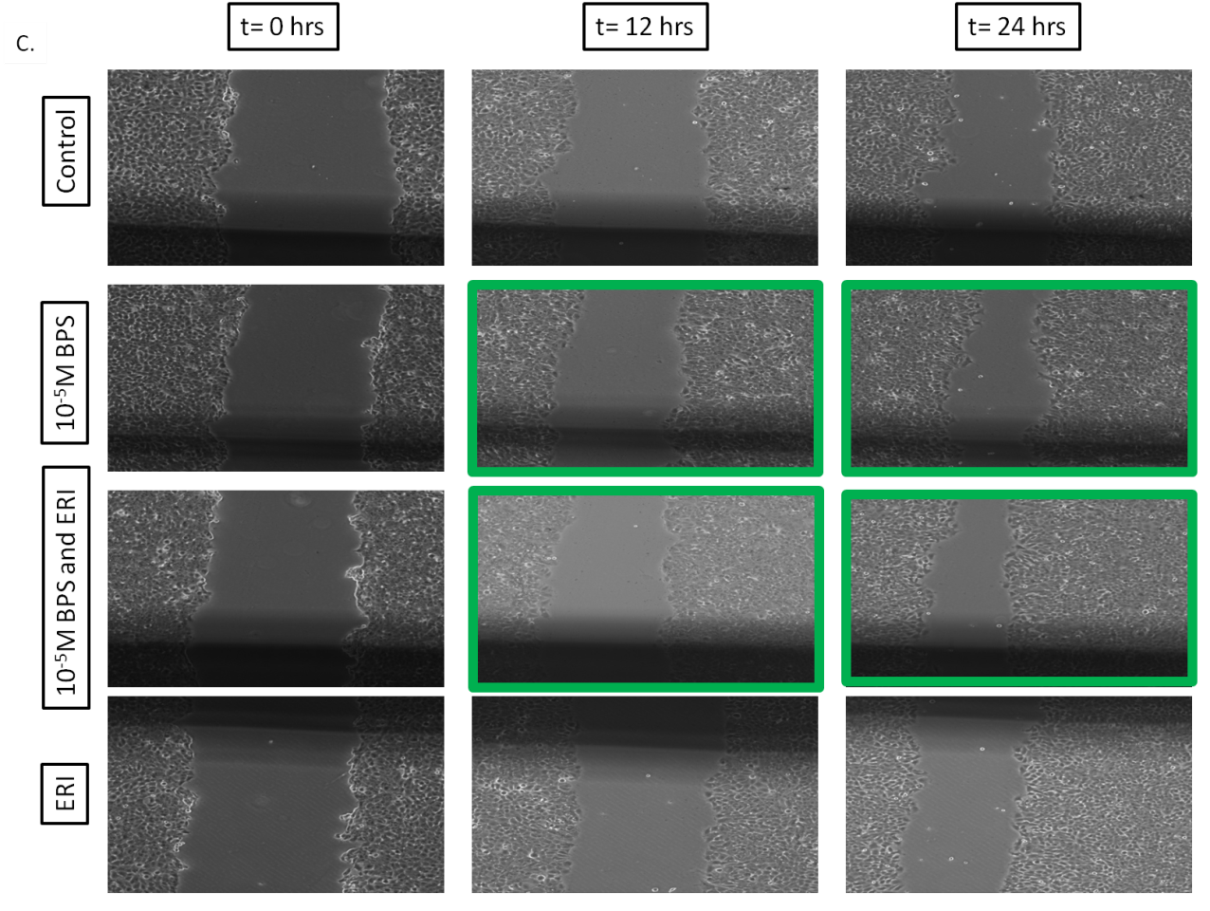
**

**Supporting Figure 6.** Representative figures of MCF-7 cells following treatment with the functional and exposure doses of bisphenol A (BPA) (A), bisphenol F (BPF) (B) and bisphenol S (BPS) (C) ± estrogen receptor inhibitor (ERI) for 24 and 48 hrs

Figures were captured using light microscope (40× magnification).

**
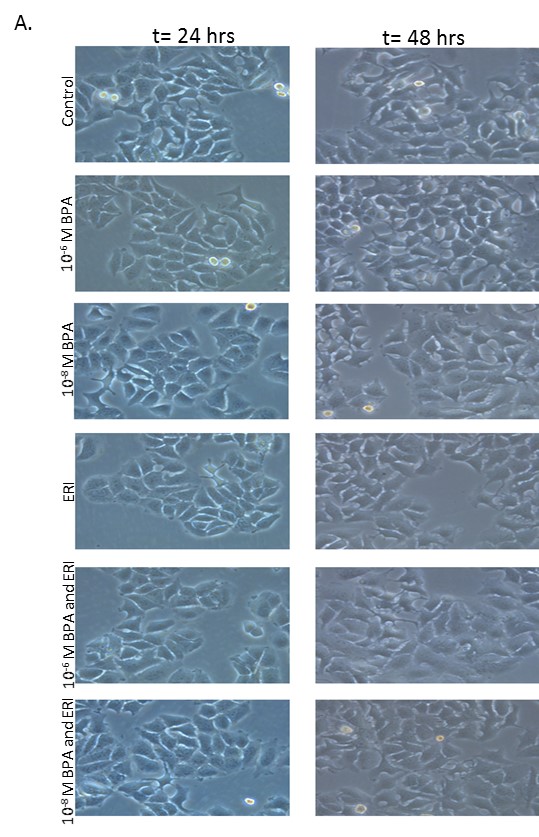
**

**
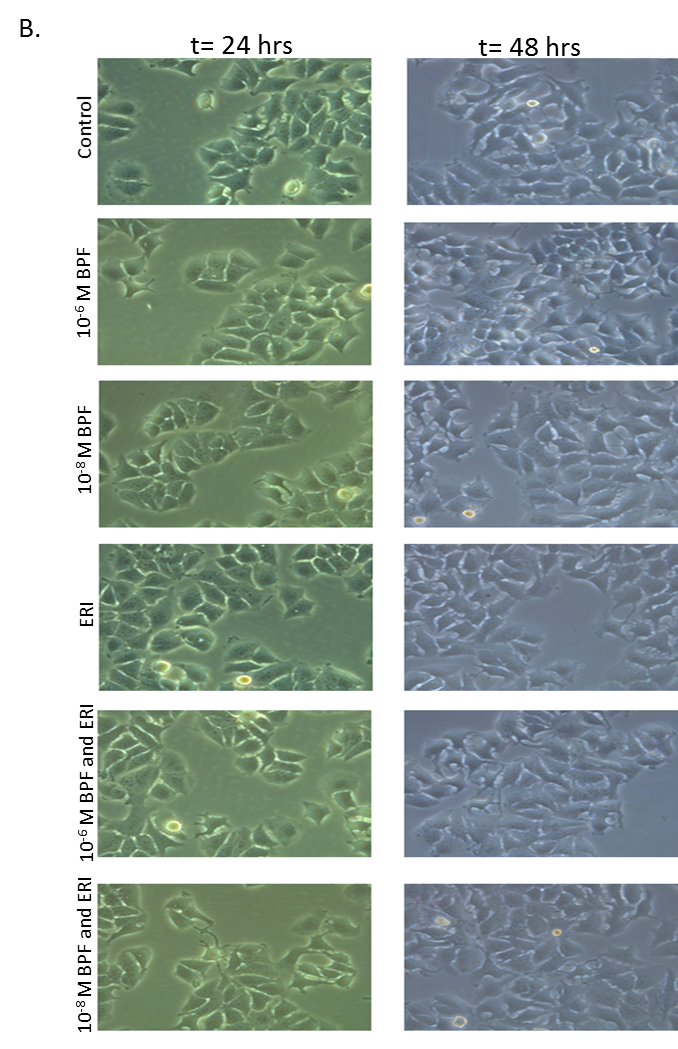
**


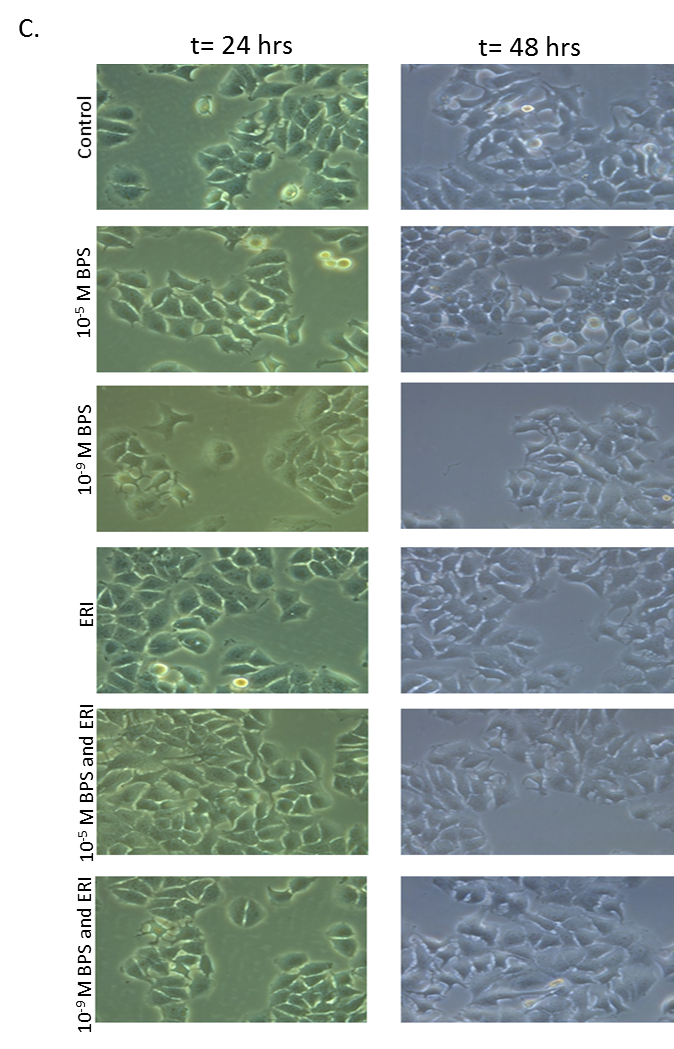


**Supporting Figure 7.** Metabolic activity (MTT assay) of MDA-MB-231 cells following treatment with different doses of bisphenol A (BPA), bisphenol F (BPF) and bisphenol S (BPS) ± estrogen receptor inhibitor (ERI) for 24hrs (A1, B1, C1), 48 hrs (A2, B2, C2) and 72 hrs (A3, B3, C3), respectively

Metabolic activity was calculated as % relative to control, and data are presented as mean + standard error of the mean (SEM) of at least three independent trials. Comparisons were performed between each treatment condition and control using one-way analysis of variance (ANOVA) followed by Dunnett *post-hoc* test (* for p < 0.05 and ** for p < 0.001), and between the same treatment condition ± ERI using one-way ANOVA followed by Tukey HSD (honestly significant difference) *post-hoc* test.


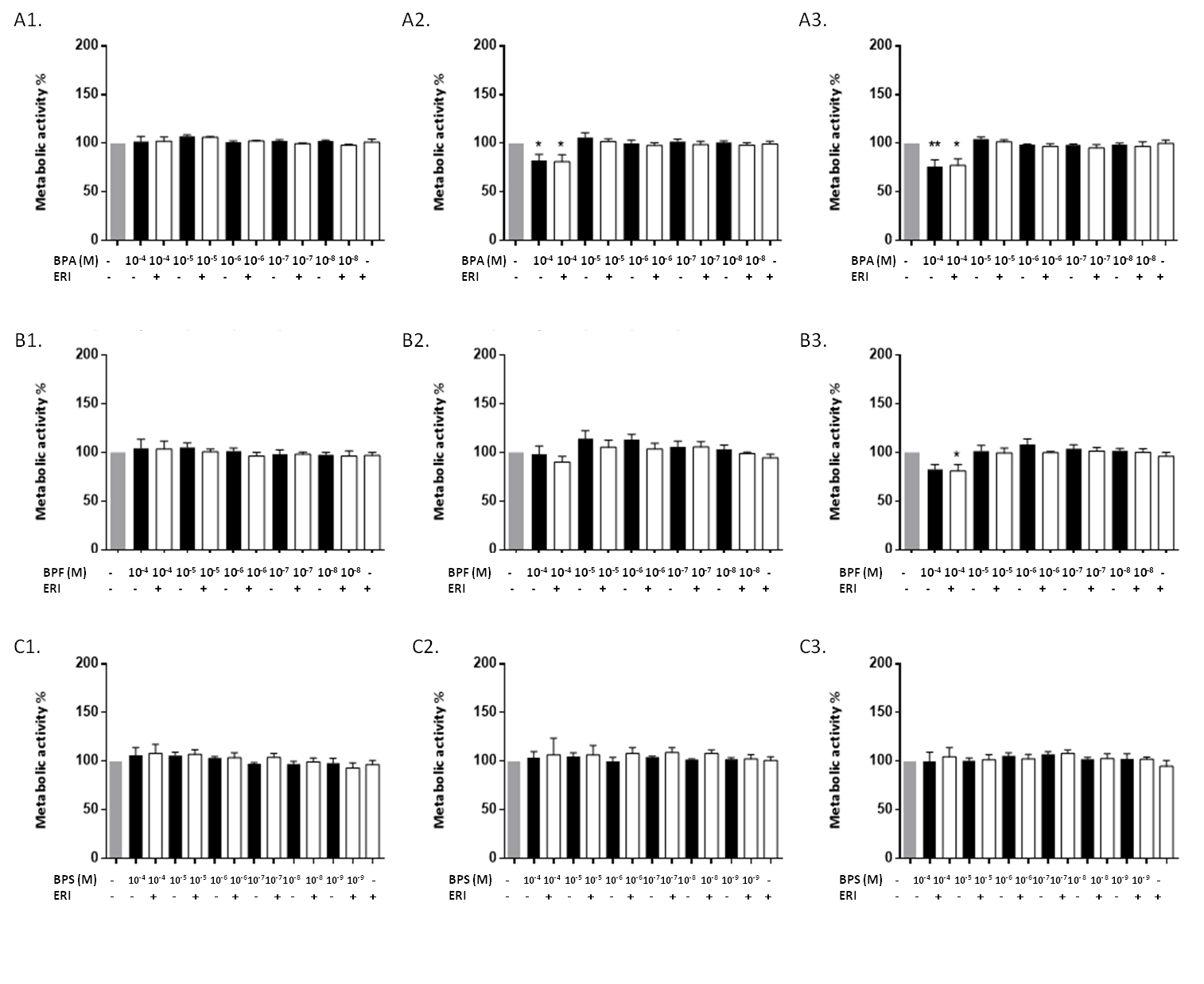


**Supporting Figure 8.** Cell viability (Trypan blue assay) of MDA-MB-231 cells following treatment with different doses of bisphenol A (BPA), bisphenol F (BPF) and bisphenol S (BPS) ± estrogen receptor inhibitor (ERI) for 24 hrs (A1, B1, C1), 48 hrs (A2, B2, C2), and 72 hrs (A3, B3, C3), respectively

Cell viability was calculated as % relative to control, and data are presented as mean + standard error of the mean (SEM) of at least three independent trials. Comparisons were performed between each treatment condition and control using one-way analysis of variance (ANOVA) followed by Dunnett *post-hoc* test (* for p < 0.05 and ** for p < 0.001), and between the same treatment condition ± ERI using one-way ANOVA followed by Tukey’s HSD (honestly significant difference) *post-hoc* test.


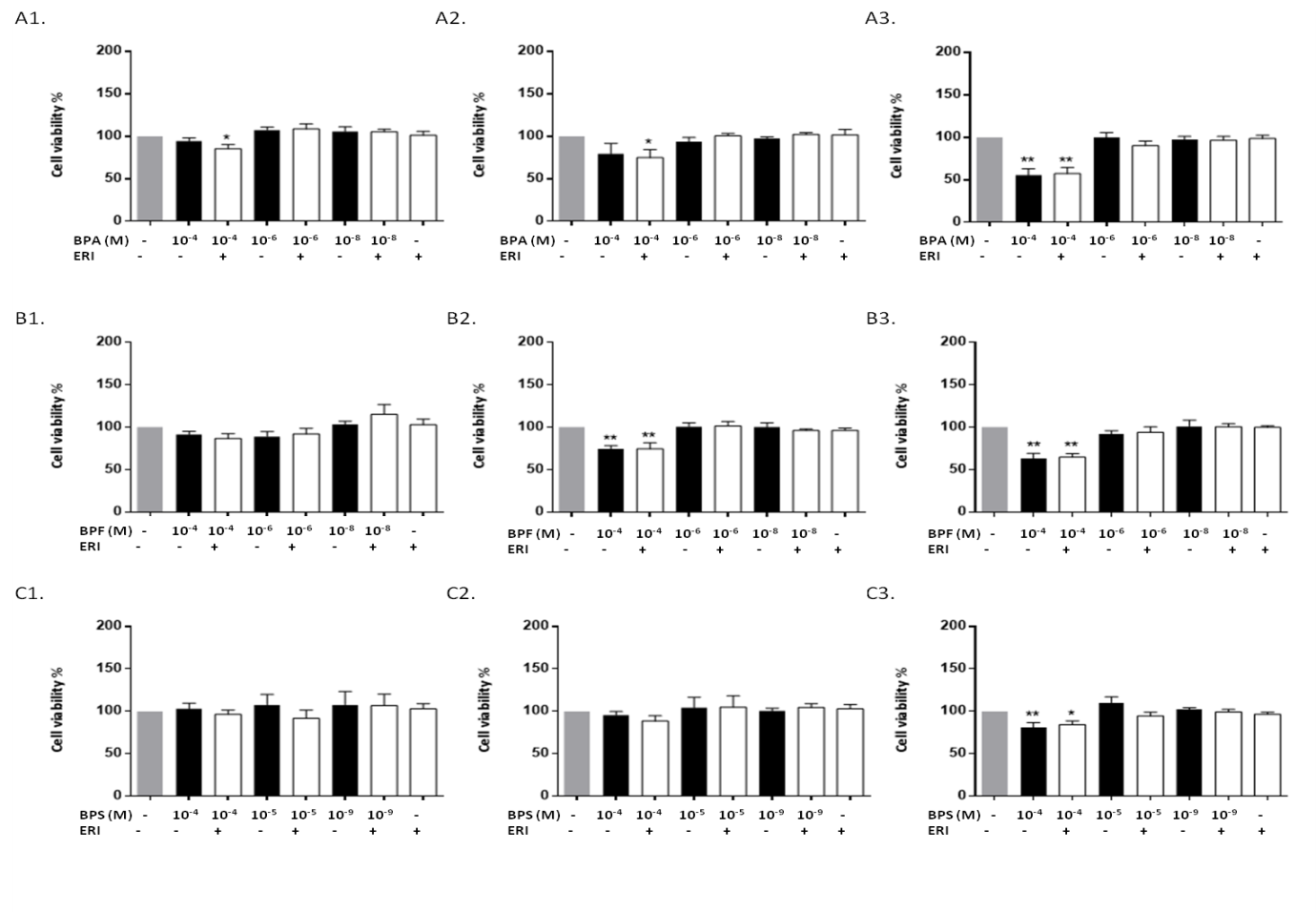


**Supporting Figure 9.** Migration (scratch assay) of MDA-MB-231 cells depicted as mean distance travelled ± standard error of the mean (SEM) over time course of treatment with exposure and functional doses of bisphenol A (BPA) (A), bisphenol F (BPF) (B) and bisphenol S (BPS) (C) ± estrogen receptor inhibitor (ERI)

Data are presented as mean distance travelled (arbitrary unit) ± SEM at time points 0, 2, 4, 6, 8, 10, 12 and 24 hrs in at least three independent trials. Comparisons were performed at every time point between each treatment condition and control using two-way analysis of variance (ANOVA) followed by Dunnett *post-hoc* test, and between the same treatment condition in the presence and absence of ERI using two-way ANOVA followed by Tukey’s HSD (honestly significant difference) *post-hoc* test.


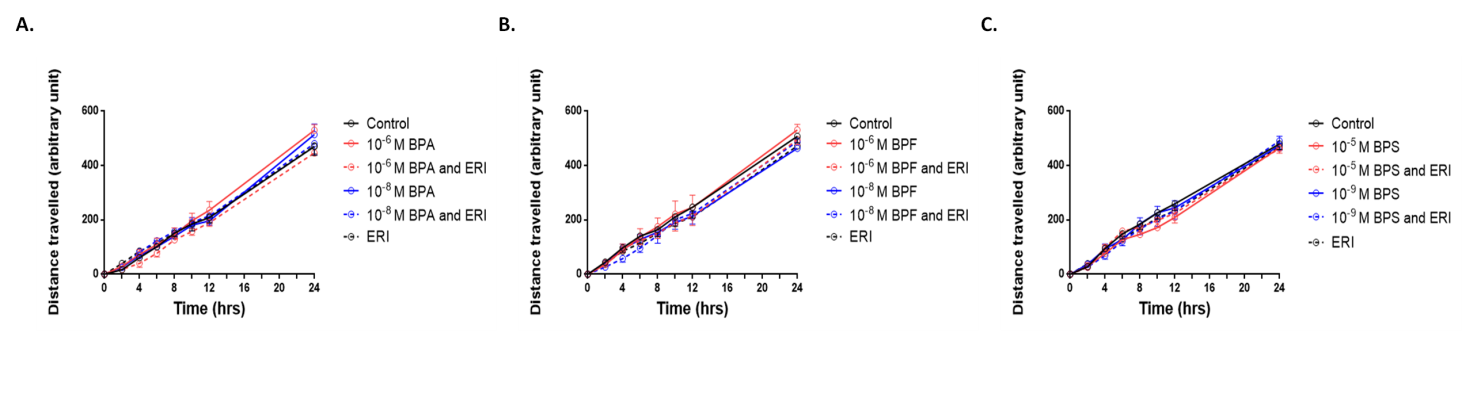


**Supporting Figure 10.** RNA expression and enzymatic activity of DNA methylation (**Panel A**) and demethylation enzymes (**Panel B**) in MCF-7 cells after 24 hrs of treatment with exposure and functional doses of bisphenol A (BPA), bisphenol F (BPF) and bisphenol S (BPS) with or without estrogen receptor inhibitor (ERI).

RNA expression and enzymatic activity were calculated as relative to control, and are presented as mean + standard error of the mean (SEM) of at least three independent trials. Comparisons were performed between each treatment condition and control using one-way analysis of variance (ANOVA) followed by Dunnett *post-hoc* test (* for p < 0.05 and ** for p < 0.001), and between the same treatment condition ± ERI using one-way ANOVA followed by Tukey’s HSD (honestly significant difference) *post-hoc* test (# for p < 0.05 and ## for p < 0.001).

**Panel A**. RNA expression of DNA methylation enzymes (DNMT1, DNMT3a, DNMT3b)and enzymatic activity of DNMTs in MCF-7 cells after 24 hrs of treatment with exposure and functional doses of BPA (A1, A2, A3, A4), BPF (B1, B2, B3, B4) and BPS (C1, C2, C3, C4), respectively.


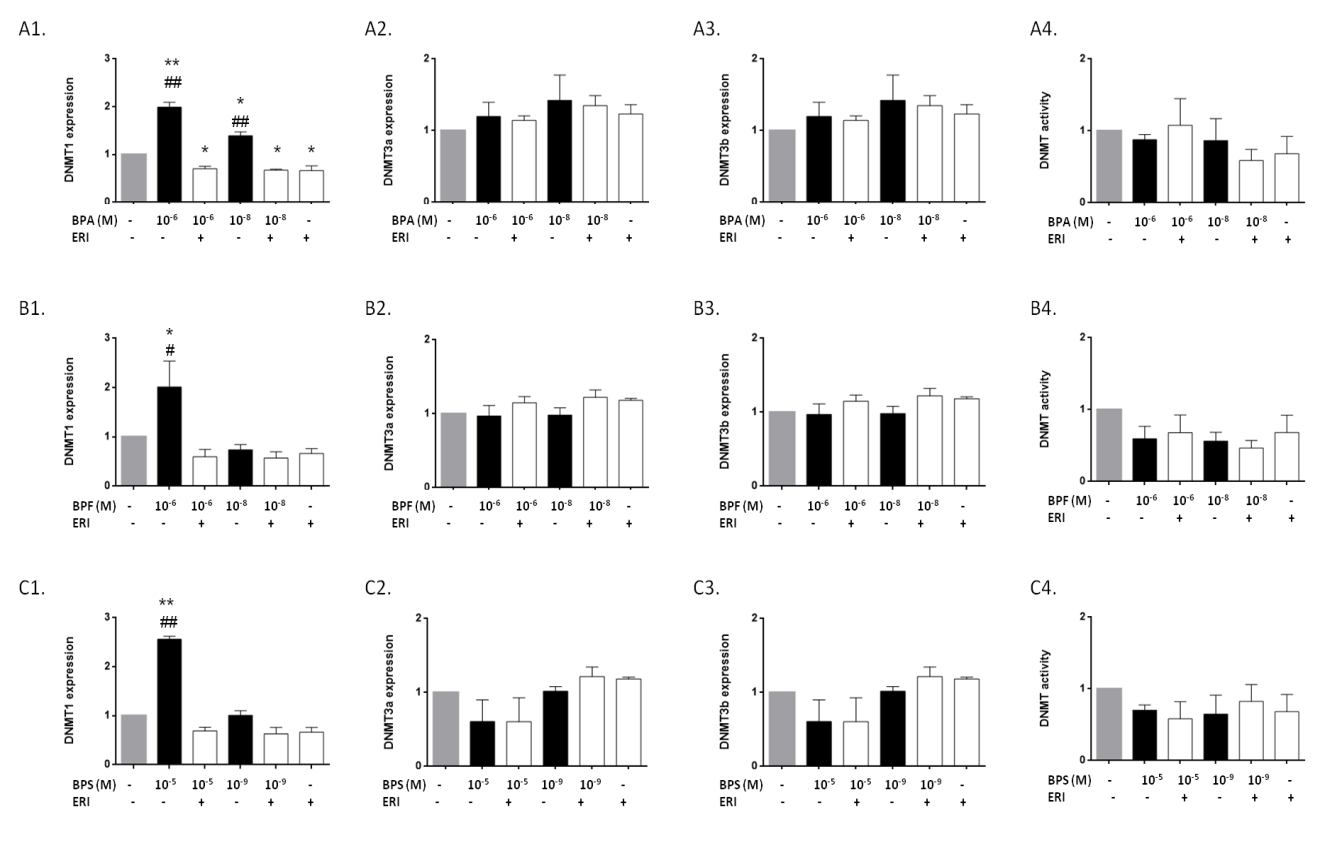


**Panel B**. RNA expression of DNA demethylation enzymes (TET1, TET2, TET3) and enzymatic activity of TETs in MCF-7 cells after 24 hrs of treatment with exposure and functional doses of BPA (A1, A2, A3, A4), BPF (B1, B2, B3, B4) and BPS (C1, C2, C3, C4), respectively


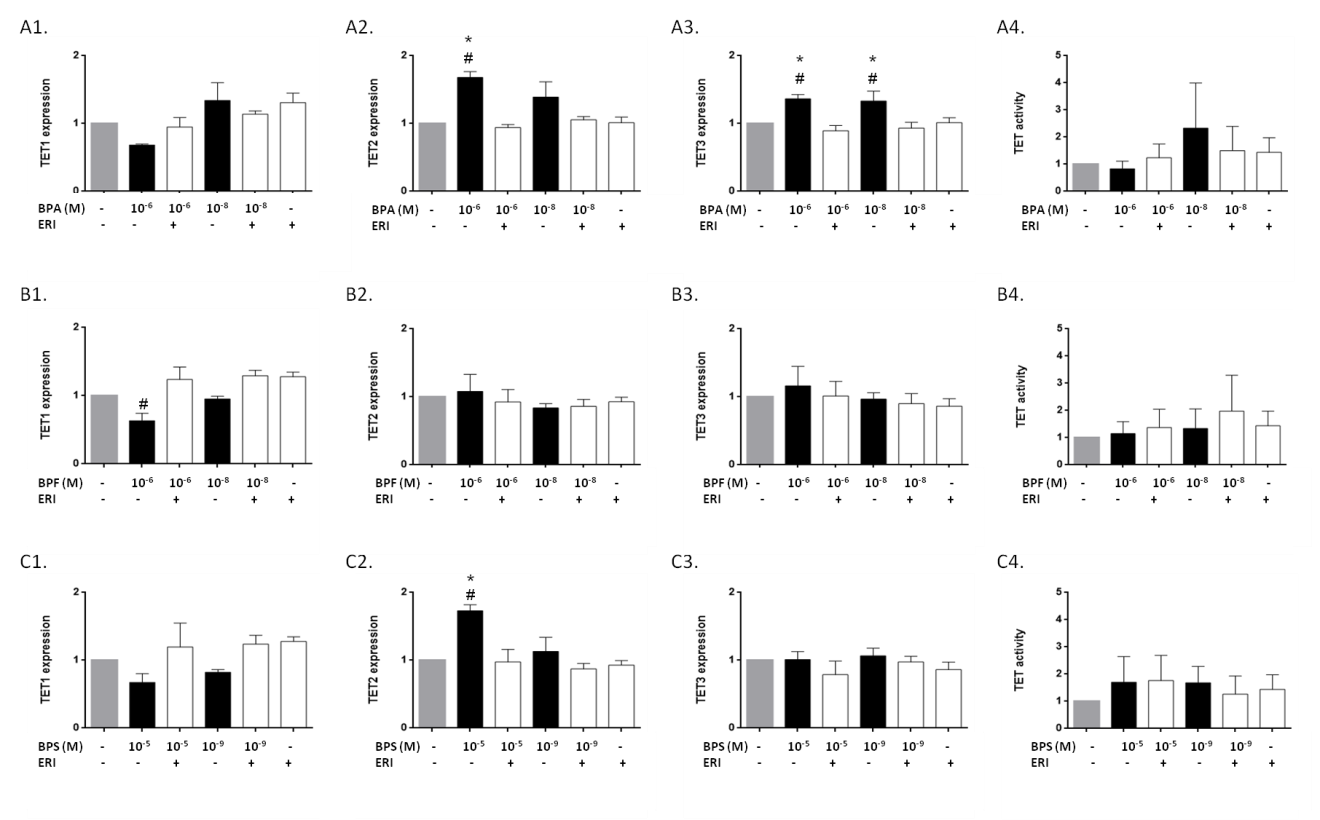


**Supporting Figure 11.** Global DNA methylation represented as DNA methylation % at 1^st^ CpG site of *LINE-1* gene in MCF-7 cells after treatment with functional doses of bisphenol A (BPA) (A1, A2), bisphenol F (BPF) (B1, B2) or bisphenol S (BPS) (C1, C2) with or without estrogen receptor inhibitor (ERI) for 24 and 48 hrs, respectively

Data are presented as mean + standard error of the mean (SEM) of three independent trials. Comparisons were performed between each treatment condition and corresponding control using one-way analysis of variance (ANOVA) followed by Dunnett *post-hoc* test, and between the same treatment condition ± ERI using one-way ANOVA followed by Tukey HSD (honestly significant difference) *post-hoc* test (^#^p-value < 0.05).

**
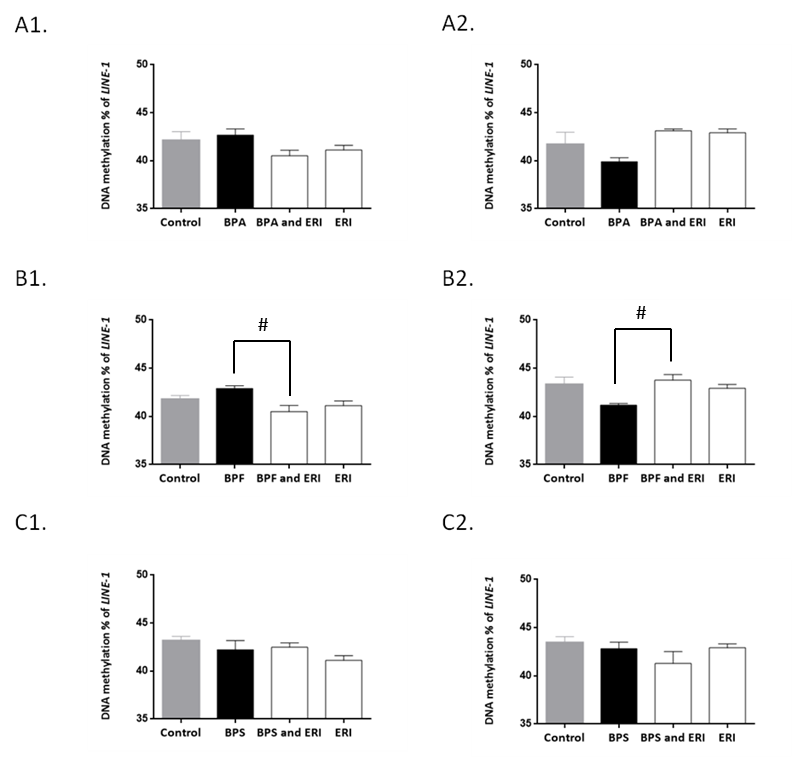
**

**Supporting Figure 12.** qq plots and lambda values for analysis of differentially methylated probes (DMPs) in MCF-7 cells treated for 48 hrs with functional doses of bisphenol A (BPA), bisphenol F (BPF), bisphenol S (BPS) with or without estrogen receptor inhibitor (ERI) or ERI alone when compared to control

**
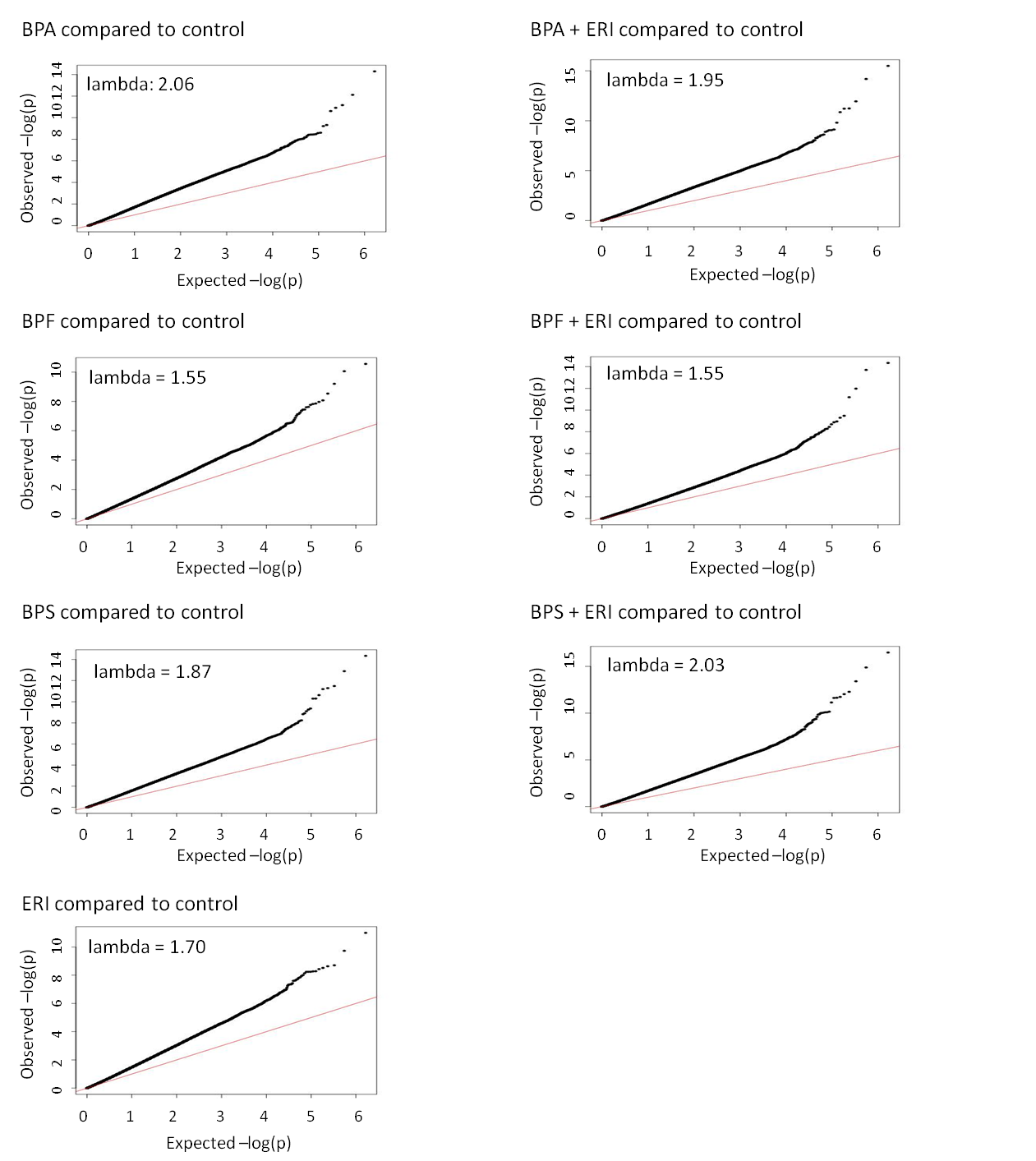
**

**Supporting Figure 13.** Differentially methylated regions (DMRs) and genes encompassing DMRs in MCF-7 cells treated for 48 hrs with the functional doses of bisphenol A (BPA), bisphenol F (BPF) and bisphenol S (BPS) when compared to control

Venn diagrams of DMRs and genes encompassing them of BPA, BPF and BPS are shown in A1-A3 and B1-B3, respectively.

Pathways of genes encompassing DMRs of BPA, BPF and BPS were detected based on KEGG pathway database using Enrichr (http://amp.pharm.mssm.edu/Enrichr/) and shown in C.

Pathways of genes with DMRs of BPA were compared to those with differentially methylated probes (DMPs) of BPA and shown in D1, and common pathways between BPA-related DMPs and DMRs are listed in D2.No figure was drawn for BPF and BPS, because their DMR-derived genes were not significantly involved in any pathway (as shown in C).

**
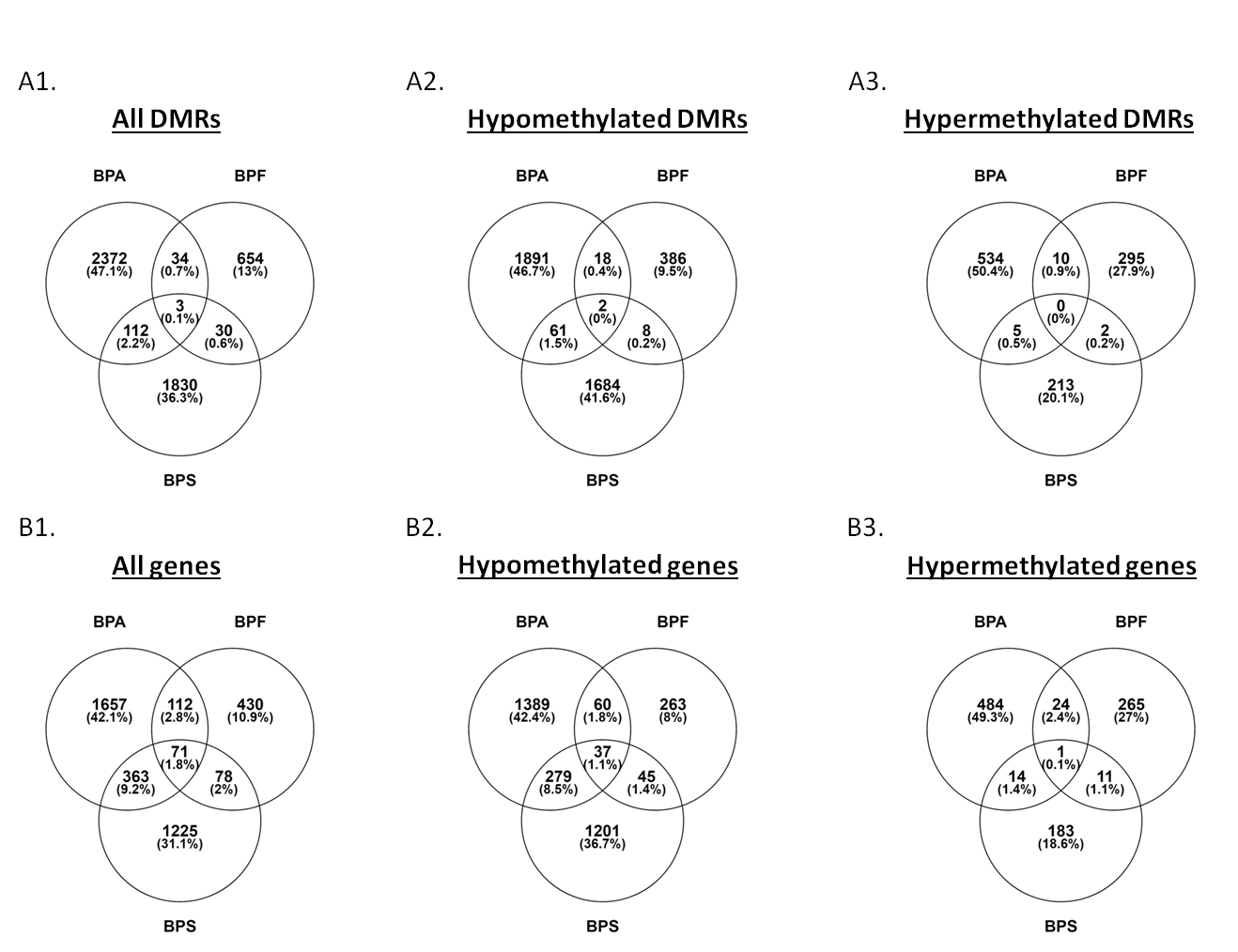
**


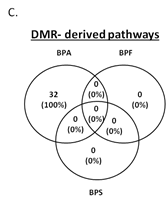


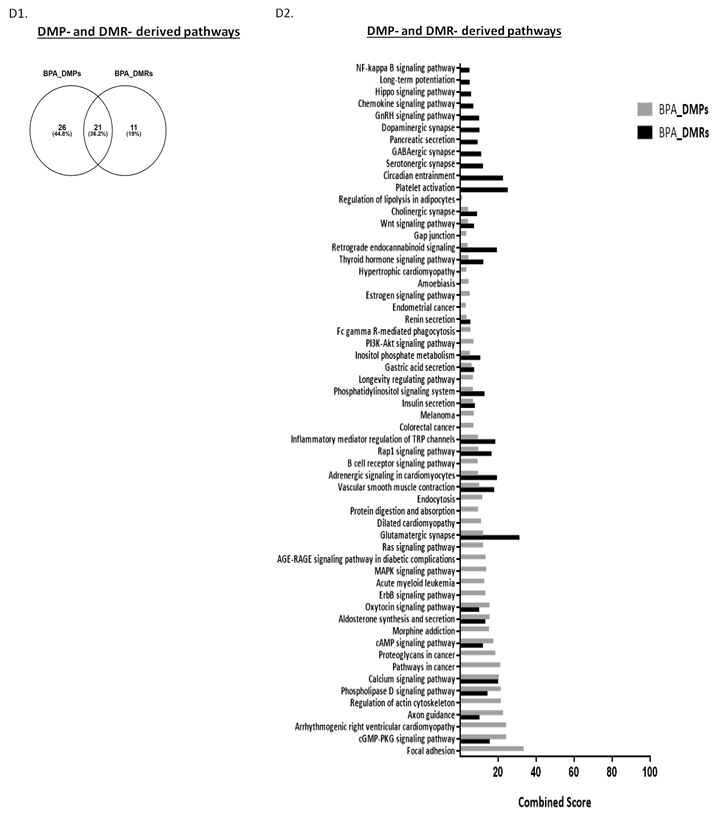


**Supporting Figure 14.** Comparison of the genomic distribution of the differentially methylated probes (DMPs) and differentially methylated regions (DMRs) in MCF-7 cells treated for 48 hrs with the functional doses of bisphenol A (BPA) (A1 and A2), bisphenol F (BPF) (B1 and B2) and bisphenol S (BPS) (C1 and C2) (in black) with the genomic distribution of randomly selected probes and regions tested by the Infinium MethylationEPIC microarray (in grey)

**
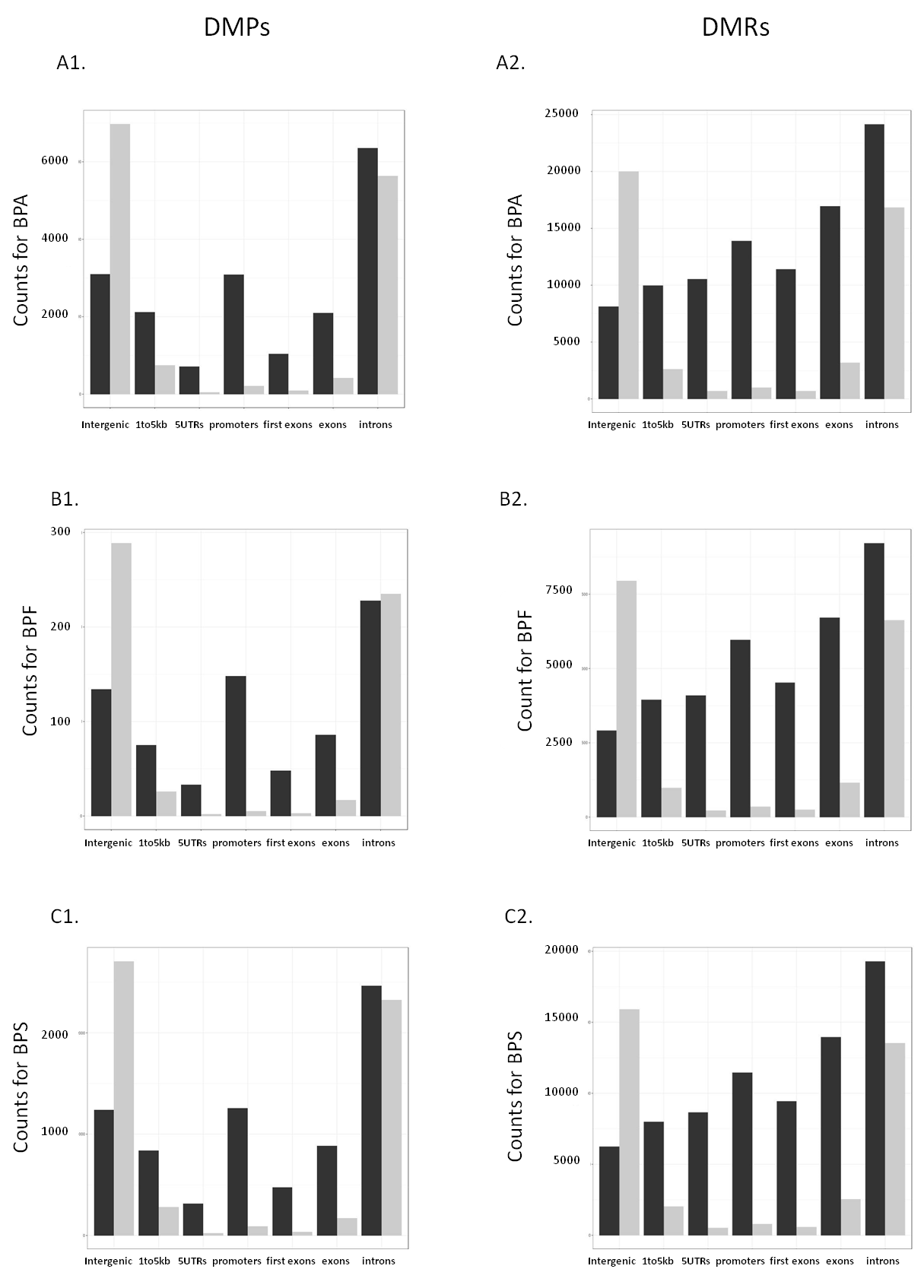
**

**Supporting Figure 15.** Validation of the Infinium MethylationEPIC microarray results using pyrosequencing

Pearson correlation analysis showing strong correlation between array and pyrosequencing results for three CpGs, one for each of the three bisphenols.

**
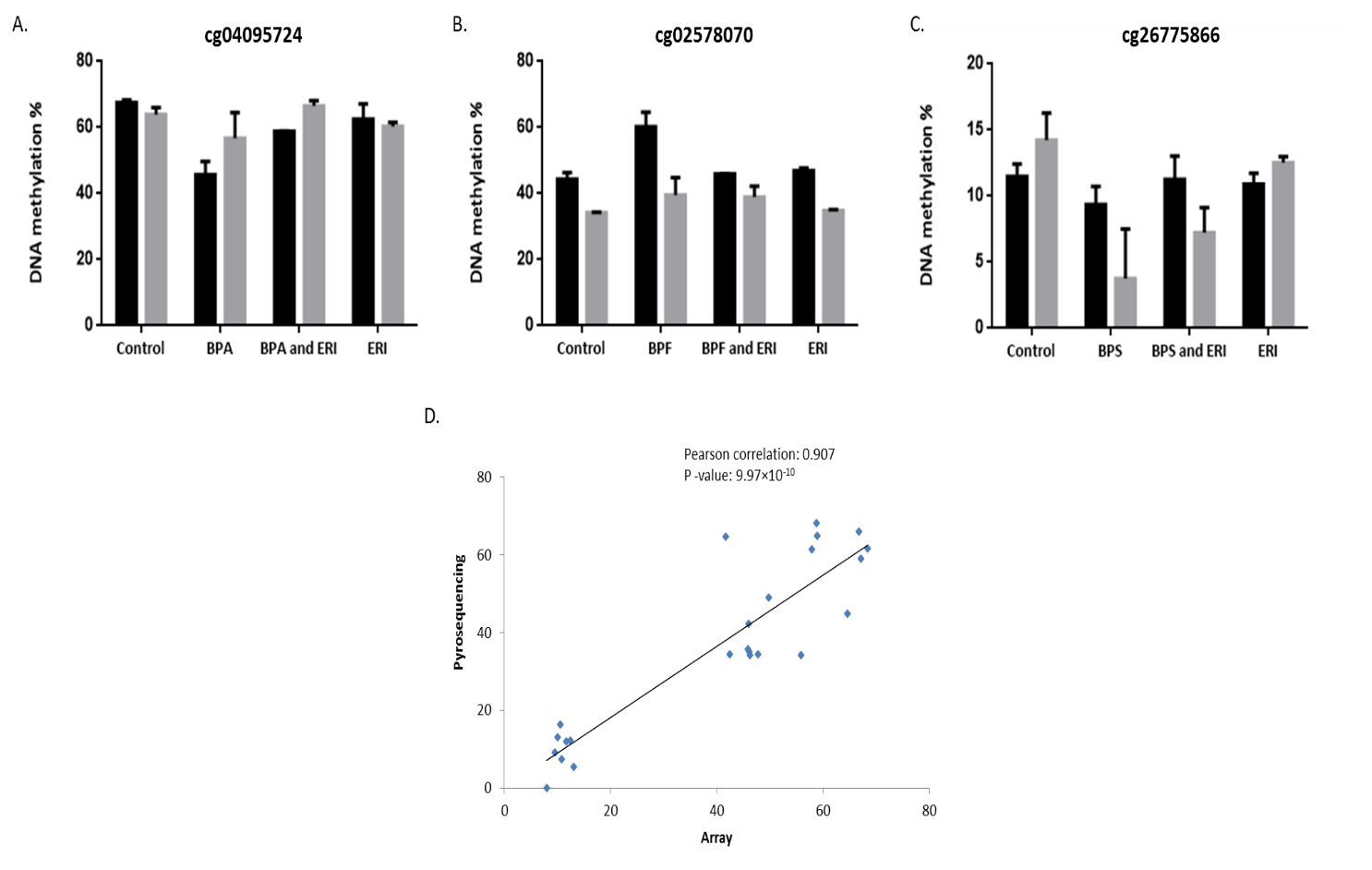
**

**Supporting Figure 16.** Venn diagrams of differentially methylated regions (DMRs) and genes encompassing them in MCF-7 cells treated for 48 hrs with the functional doses of bisphenol A (BPA) (A1-A6), bisphenol F (BPF) (B1-B6) and bisphenol S (BPS) (C1-C6) treatment conditions with or without estrogen receptor inhibitor (ERI) and ERI alone when compared to control


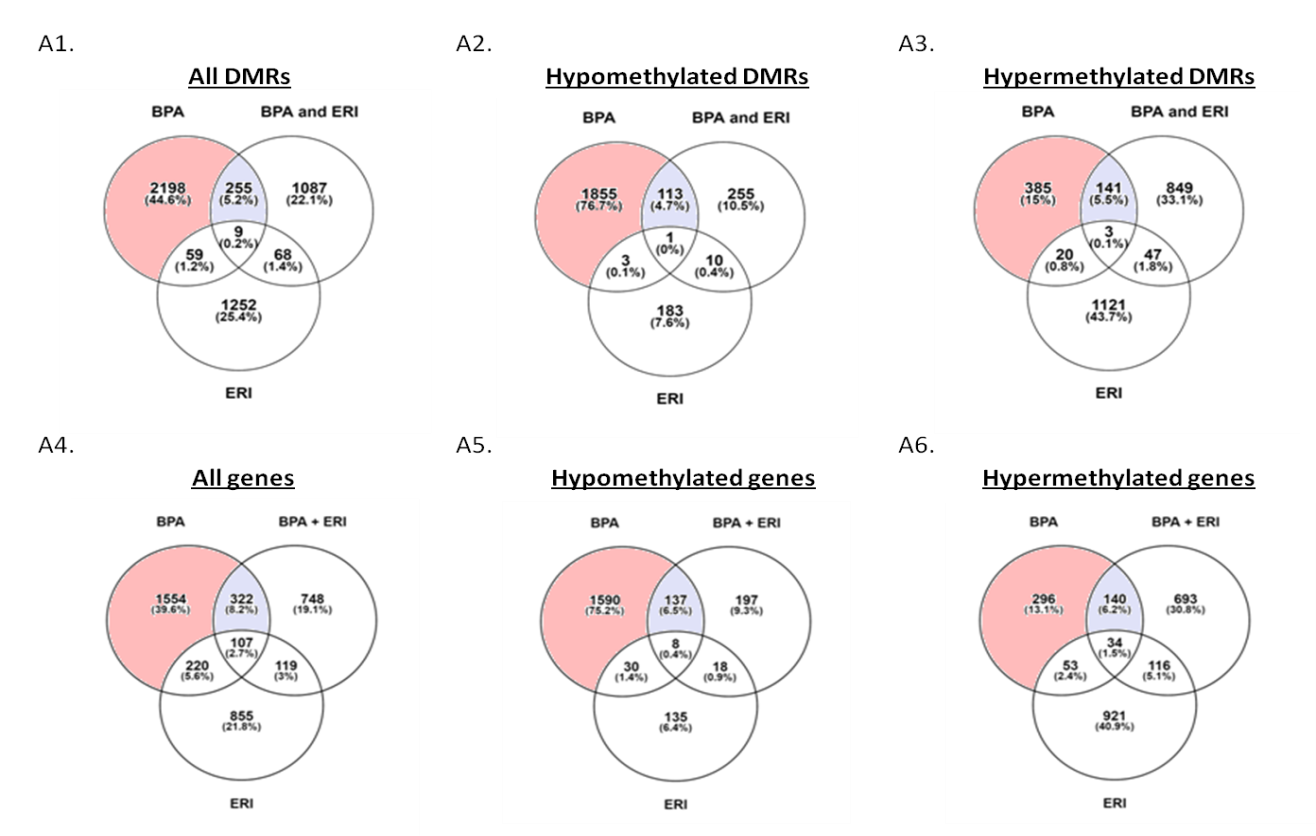


**
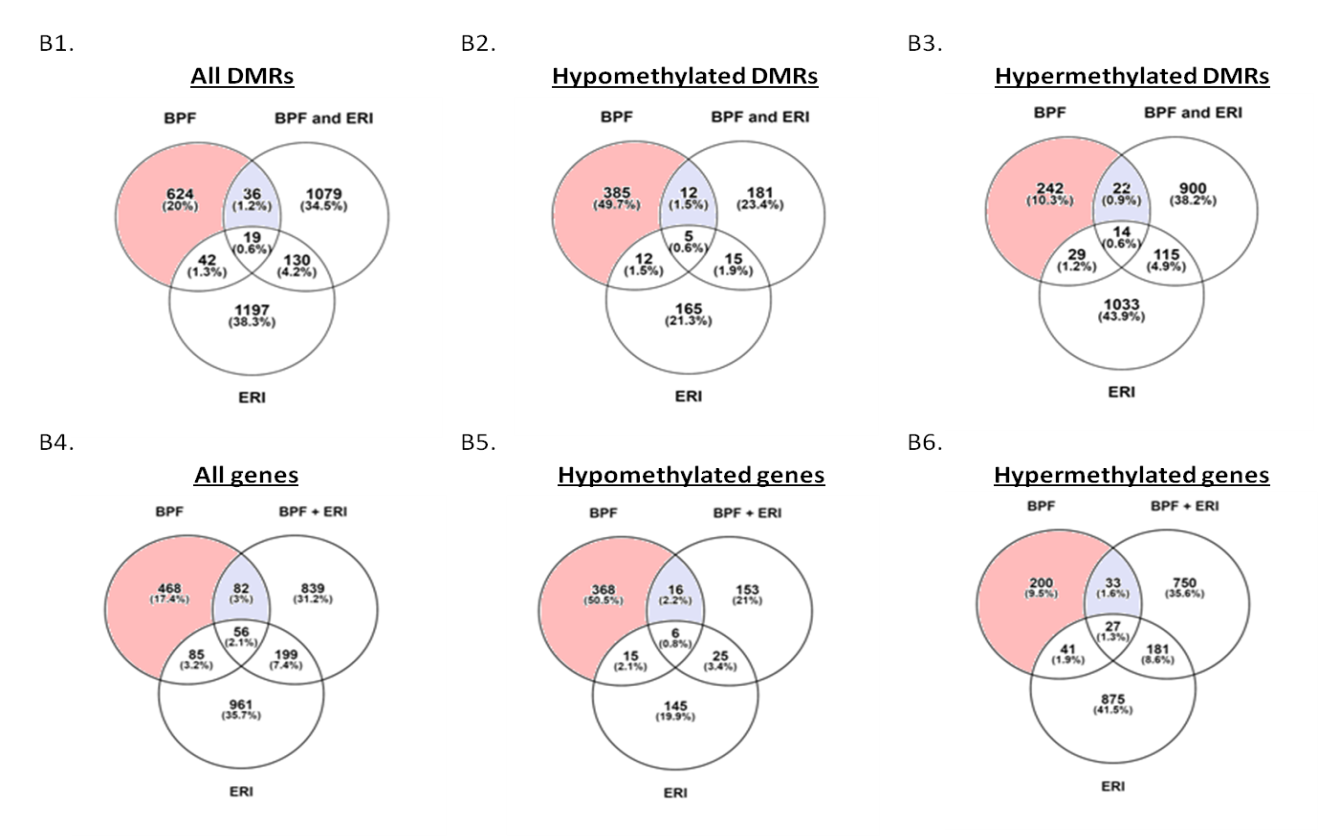
**


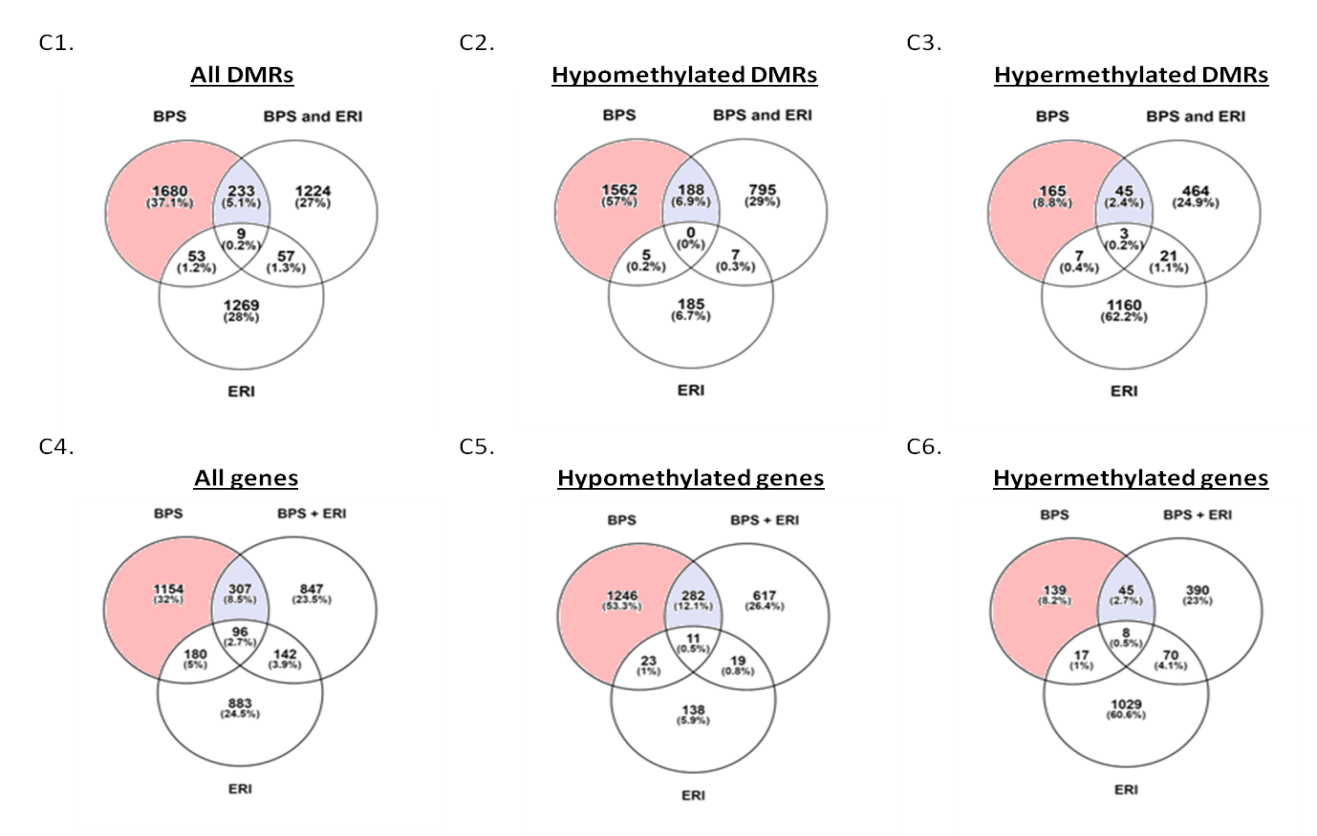

Supplement: Supplementary file 1 — Supporting Figures S1–S16. (DOCX 8.22 kb) [file 13148_2019_725_MOESM1_ESM.docx]
