# Supplementary material for: DNA methylome-wide alterations associated with estrogen receptor-dependent effects of bisphenols in breast cancer
Source: Clin Epigenetics. 2019 Oct 10;11:138. doi: 10.1186/s13148-019-0725-y (PMC6785895; doi:10.1186/s13148-019-0725-y)
Supplement: Supplementary file 2 — Supporting Tables S1–S4. (DOCX 24.9 kb) [file 13148_2019_725_MOESM2_ESM.docx]

**Supporting Table 1.** Primers used in the different assays

|  | **Primers** | **Reference** |
| --- | --- | --- |
| ***LINE-1 pyrosequencing*** | | Daskalos et al. (2009)^1^ |
| Forward primer | 5’ btn-TAG GGAGTGTTAGATAGTGG 3’ |  |
| Reverse primer | 5’ AACTCCCTAACCCCTTAC 3’ |  |
| Sequencing primer | 5’ CAAATAAAACAATACCTC 3’ |  |
| **Gene expression** | |  |
| ***DNA methyltransferase 1 (DNMT1)*** | | Sheng et al. (2013)^2^ |
| Forward primer | 5’ AAACCCCTTTCCAAACCTCG 3’ |  |
| Reverse primer | 5’ CTGGTGCTTTTCCTTGTAATCC 3’ |  |
| ***DNA methyltransferase 3A (DNMT3A)*** | | Sheng et al. (2013)^2^ |
| Forward primer | 5' CCAAGTTCAGCAAAGTGAGGAC3' |  |
| Reverse primer | 5' TGGACTGGGAAACCAAATACC3' |  |
| ***DNA methyltransferase 3B (DNMT3B)*** | | Sheng et al. (2013)^2^ |
| Forward primer | 5' TCCCAGCTCTTACCTTACCATC 3' |  |
| Reverse primer | 5' ATCTCCACTGTCTGCCTCCA 3' |  |
| ***Ten-eleven translocation 1 (TET1)*** | | Guidotti et al. (2012)^3^ |
| Forward primer | 5’-CCCGGGCTCCAAAGTTGTG 3’ |  |
| Reverse primer | 5’-GCAGGAAACAGAGTCATTGGTCCT 3’ |  |
| ***Ten-eleven translocation 2 (TET2)*** | | Guidotti et al. (2012)^3^ |
| Forward primer | 5’-GAAAGGAGACCCGACTGCAACTG 3’ |  |
| Reverse primer | 5’-GCAGCTCAGTCCCTTACTGCTC 3’ |  |
| ***Ten-eleven translocation (TET3)*** | | Guidotti et al. (2012)^3^ |
| Forward primer | 5’-CAGTGGCTTCTTGGAGTCACCTC 3’ |  |
| Reverse primer | 5’-GGATGGCTTTCCCCTTCTCTCC 3’ |  |
| ***ß2-microglobulin*** |  | Sheng et al. (2013)^2^ |
| Forward primer | 5' TGCTGTCTCCATGTTTGATGTATCT3' |  |
| Reverse primer | 5' TCTCTGCTCCCCACCTCTAAGT 3' |  |

^1^Daskalos A, Nikolaidis G, Xinarianos G, et al. Hypomethylation of retrotransposable elements correlates with genomic instability in non-small cell lung cancer. *Int J Cancer.* 2009;124(1):81-87

^2^Sheng W, Qian Y, Wang H, et al. Association between mRNA levels of DNMT1, DNMT3A, DNMT3B, MBD2 and LINE-1 methylation status in infants with tetralogy of Fallot. *Int J Mol Med.* 2013;32(3):694-702

^3^Guidotti A, Dong E, Gavin DP, et al. DNA methylation/demethylation network expression in psychotic patients with a history of alcohol abuse. *Alcohol Clin Exp Res.* 2013;37(3):417-424.

**Supporting Table 2.** Primers used for validation of DNA methylome-wide array results with pyrosequencing

|  | **Primers** |
| --- | --- |
| **cg04095724 (BPA)** | |
| Forward primer | 5’ TTAGGTAAGAGGGTGGGTGTT 3’ |
| Reverse primer | 5’ ACCCCTTACTTAACTCCCTC 3’ |
| Sequencing primer | 5’ TAGTTTTTTTAGTAGGGT 3’ |
| **cg02578070 (BPF)** | |
| Forward primer | 5’ GGGGGTTGATTAGTGTYGTYG 3’ |
| Reverse primer | 5’ RCCRTTCAAAAACCTACCCCC 3’ |
| Sequencing primer | 5’ GTTGTTATTGGTGGGG 3’ |
| **cg26775866 (BPS)** | |
| Forward primer | 5’ GTGAGTGAATGGGAGGGT 3’ |
| Reverse primer | 5' ACCCCAACTTCAACCCATC 3' |
| Sequencing primer | 5' TTAAGTATTTGTTGGTTTA 3' |

**Supporting Table 3.** Number of differentially methylated probes (DMPs) and differentially methylated regions (DMRs) before and after filtration and their corresponding genes in MCF-7 cells treated for 48 hrs with functional doses of BPA, BPF and BPS with or without ERI when compared to control

| **Treatment**^†^ | **DMPs** | | | | **DMRs** | | | |
| --- | --- | --- | --- | --- | --- | --- | --- | --- |
|  | Before filtration | After filtration  (hypo/hyper) | Genes (hypo/hyper) | Max IΔßI (%) | Before filtration | After filtration (hypo/hyper) | Genes (hypo/hyper) | Max  IΔßI (%) |
| **BPA** | 13366 | 6574 (4469/2105) | 3622 (2682/1252) | 22.77 | 38193 | 2521 (1972/549) | 2203 (1765/523) | 13.43 |
| **BPA + ERI** | 9534 | 4225 (1765/2460) | 2478 (1091/1559) | 25.06 | 34076 | 1419 (379/1040) | 1296 (360/983) | 18.32 |
| **BPF** | 549 | 190 (83/107) | 121 (56/65) | 25.47 | 15151 | 721 (414/307) | 691 (405/301) | 10.92 |
| **BPF + ERI** | 1243 | 598 (266/332) | 409 (172/243) | 25.58 | 17214 | 1264 (213/1051) | 1176 (200/991) | 15.77 |
| **BPS** | 5309 | 2361 (1585/776) | 1495 (1032/519) | 33.35 | 30559 | 1975 (1755/220) | 1737 (1562/209) | 19.5 |
| **BPS + ERI** | 1097 | 555 (281/274) | 401 (206/198) | 18.77 | 37478 | 1523 (990/533) | 1392 (929/513) | 12.05 |
| **ERI** | 2829 | 1185 (332/853) | 764 (208/567) | 34.83 | 23954 | 1388 (197/1191) | 1301 (191/1124) | 16.17 |

^†^ Treatment conditions were compared to control using robust linear model (RLM).

Hypo: hypomethylated; hyper: hypermethylated

IΔßI: absolute value of the difference of filtered methylation % between treatment and control

**Supporting Table 4.** CpG sites similarly dysregulated in MCF-7 cells treated for 48 hrs with functional doses of BPA and BPS when compared to control and in 595 ER+ tumor tissues when compared to 124 normal-adjacent tissues in breast cancer patients from The Cancer Genome Atlas (TCGA) database

| **CpG sites** | **Δß (ß_tumor_ - ß_normal_)** | **Δß (ß_BPA/BPS_ - ß_control_)** | **Methylation**  **change** | **Gene** | **Gene name** |
| --- | --- | --- | --- | --- | --- |
| **Common CpG sites between BPA and breast cancer** | | | | | |
| cg10861751 | -21.53 | -4.51 | Hypo | *RGS1* | *Regulator of G protein signaling 1* |
| cg09147827 | -18.09 | -11.34 | Hypo | *SERPINA6* | *Serpin Family A Member 6* |
| cg02097420 | -16.85 | -5.36 | Hypo | *HRG* | *Histidine Rich Glycoprotein* |
| cg09448875 | -16.85 | -5.55 | Hypo | *ABCC2* | *ATP Binding Cassette Subfamily C Member 2* |
| cg10818284 | -9.62 | -8.04 | Hypo | *SYP* | *Synaptophysin* |
| cg00077877 | -8.39 | -6.11 | Hypo | *ASAP1* | *ArfGAP With SH3 Domain, Ankyrin Repeat And PH Domain 1* |
| cg26775866 | -7.11 | -7.87 | Hypo | *PTTG1* | *Pituitary Tumor-Transforming 1* |
| cg24719601 | 9.64 | 3.61 | Hyper | *PHOX2B* | *Paired Like Homeobox 2b* |
| cg22411207 | 9.99 | 4.63 | Hyper | *MOS* | *MOS Proto-Oncogene, Serine/Threonine Kinase* |
| cg18793806 | 15.34 | 4.34 | Hyper | *ZNF514* | *Zinc Finger Protein 514* |
| cg17020834 | 34.34 | 3.26 | Hyper | *GRIA1* | *Glutamate Ionotropic Receptor AMPA Type Subunit 1* |
| **Common CpG sites between BPS and breast cancer** | | | | | |
| cg01078434 | -27.97 | -3.80 | Hypo | *MAS1L* | *MAS1 Proto-Oncogene Like, G Protein-Coupled Receptor* |
| cg09096383 | -19.94 | -13.02 | Hypo | *CSN1S1* | *Casein Alpha S1* |
| cg00474004 | -17.97 | -3.95 | Hypo | *IFNA14* | *Interferon Alpha 14* |
| cg06243556 | 7.02 | 4.32 | Hyper | *ZSCAN18* | *Zinc Finger And SCAN Domain Containing 18* |
| cg00174901 | 15.77 | 7.10 | Hyper | *PALM* | *Paralemmin* |
| cg21672276 | 18.40 | 9.71 | Hyper | *ZNF502* | *Zinc Finger Protein 502* |
| cg12876594 | 20.06 | 3.28 | Hyper | *NPR2* | *Natriuretic Peptide Receptor 2* |
| cg14614211 | 22.52 | 4.59 | Hyper | *MKX* | *Mohawk Homeobox* |
| cg01580681 | 27.10 | 6.88 | Hyper | *HAND2* | *Heart And Neural Crest Derivatives Expressed 2* |

Hypo: hypomethylated; hyper: hypermethylated; ß: methylation percentage

Note that no CpG site was similarly dysregulated between BPF and breast cancer, so BPF is not included in the table.
